# Identification of the role of DAB2 and CXCL8 in uterine spiral artery remodeling in early-onset preeclampsia

**DOI:** 10.1007/s00018-024-05212-4

**Published:** 2024-04-13

**Authors:** Yu Liu, Lili Du, Shifeng Gu, Jingying Liang, Minshan Huang, Lijun Huang, Siying Lai, Shuang Zhang, Zhaowei Tu, Wei Sun, Dunjin Chen, Jingsi Chen

**Affiliations:** 1 Department of Obstetrics and Gynecology, Guangdong Provincial Key Laboratory of Major Obstetric Diseases; Guangdong Provincial Clinical Research Center for Obstetrics and Gynecology; Guangdong-Hong Kong-Macao Greater Bay Area Higher Education Joint Laboratory of Maternal-Fetal Medicine; The Third Affiliated Hospital of Guangzhou Medical University, Guangzhou, 510150 China; 2 Department of Fetal Medicine and Prenatal Diagnosis, The Third Affiliated Hospital of Guangzhou Medical University, Guangzhou, 510150 China

**Keywords:** Early-onset preeclampsia, DAB2, CXCL8, Spiral artery remodeling

## Abstract

**Abstract:**

Aberrant remodeling of uterine spiral arteries (SPA) is strongly associated with the pathogenesis of early-onset preeclampsia (EOPE). However, the complexities of SPA transformation remain inadequately understood. We conducted a single-cell RNA sequencing analysis of whole placental tissues derived from patients with EOPE and their corresponding controls, identified DAB2 as a key gene of interest and explored the mechanism underlying the communication between Extravillous trophoblast cells (EVTs) and decidual vascular smooth muscle cells (dVSMC) through cell models and a placenta-decidua coculture (PDC) model in vitro. DAB2 enhanced the motility and viability of HTR-8/SVneo cells. After exposure to conditioned medium (CM) from HTR-8/SVneo^shNC^ cells, hVSMCs exhibited a rounded morphology, indicative of dedifferentiation, while CM-HTR-8/SVneo^shDAB2^ cells displayed a spindle-like morphology. Furthermore, the PDC model demonstrated that CM-HTR-8/SVneo^shDAB2^ was less conducive to vascular remodeling. Further in-depth mechanistic investigations revealed that C-X-C motif chemokine ligand 8 (CXCL8, also known as IL8) is a pivotal regulator governing the dedifferentiation of dVSMC. DAB2 expression in EVTs is critical for orchestrating the phenotypic transition and motility of dVSMC. These processes may be intricately linked to the CXCL8/PI3K/AKT pathway, underscoring its central role in intricate SPA remodeling.

**Graphical abstract:**

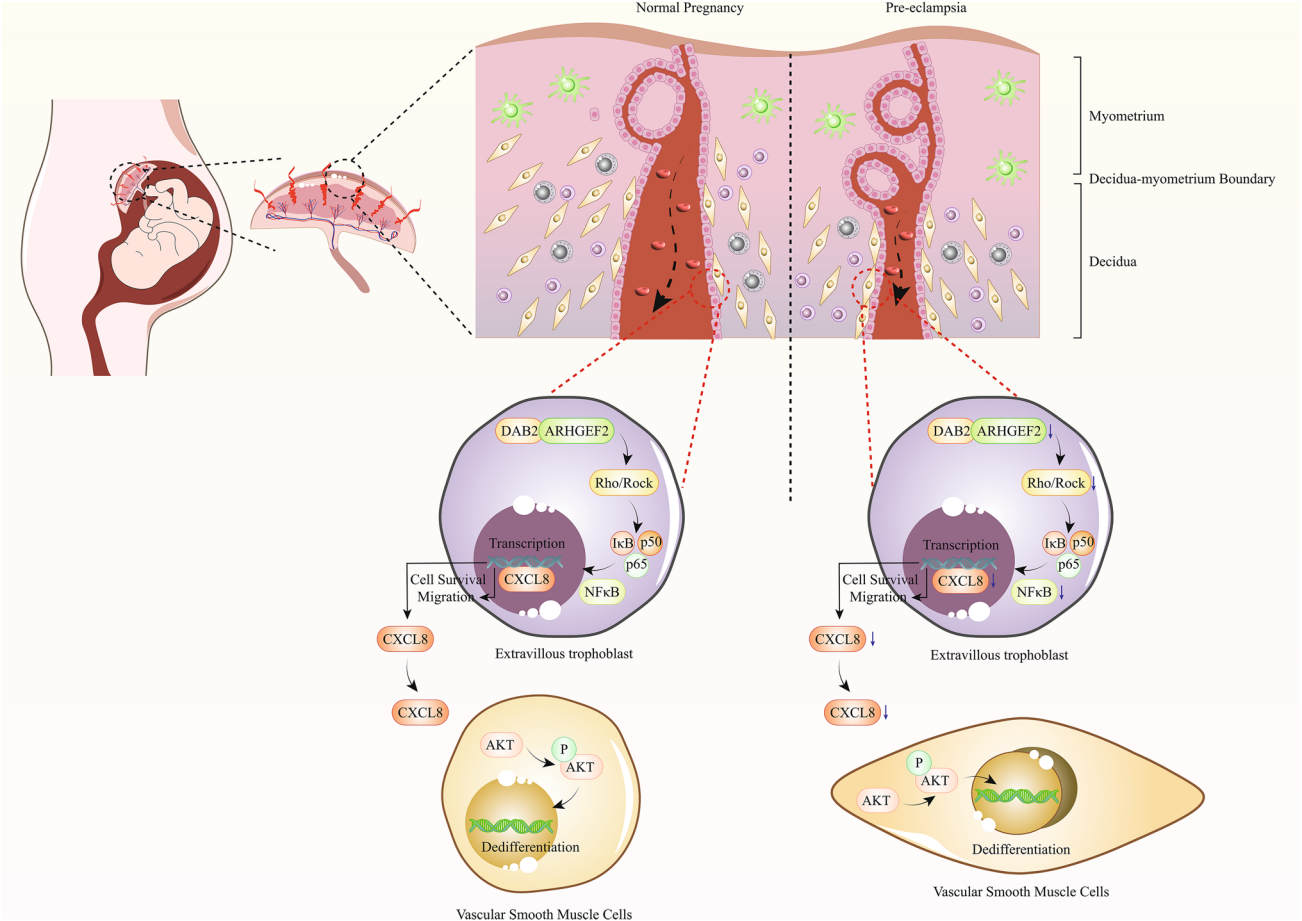

**Supplementary Information:**

The online version contains supplementary material available at 10.1007/s00018-024-05212-4.

## Introduction

Preeclampsia, new-onset hypertension and proteinuria develop after 20 weeks of pregnancy and resolve after delivery [1].This condition is characterized by maternal systemic inflammation and vascular dysfunction [2]. Early-onset preeclampsia (EOPE) is diagnosed before the 34th week of gestation [3], and often results from placental factors such as trophoblast invasion failure, endothelial dysfunction, inadequate angiogenesis, and insufficient uterine artery remodeling [4–7]. EOPE is characterized by early gestational age at onset and severe clinical symptoms. Organ damage in mothers can lead to various complications, including renal failure, HELLP syndrome (characterized by hemolysis, elevated liver enzymes, and low platelet count), thrombocytopenia, disseminated intravascular coagulation, pulmonary edema, and eclampsia (seizures). Adverse outcomes for fetuses or neonates can arise from intrauterine growth restriction (IUGR), prematurity, or placental abruption [8]. EOPE is associated with increased risks of neonatal mortality and maternal morbidity [4, 9]. Therefore, understanding the underlying mechanisms of EOPE is crucial for mitigating its pathogenesis and exploring interventions for this disease. The success of a pregnancy relies on intricate cellular interactions at the maternal–fetal interface, where spiral artery remodeling (SAR) is crucial for fetal development. Interestingly, while vessel remodeling is pathological in cardiovascular diseases such as atherosclerosis, in pregnancy, it is a highly regulated physiological process. Optimal physiological vascular remodeling involves fetal extravillous trophoblasts (EVTs) [10], yet the cellular and molecular aspects of spiral artery (SpA) remodeling have not been fully elucidated. Notably, the observed dedifferentiation, migration, and apoptosis of vascular smooth muscle cells (VSMCs) around EVT-associated vessels are hallmarks of early SpA remodeling [11]. VSMCs are not mere spectators of the remodeling process. Interactions with trophoblasts influence a range of cellular activities, including protease production [12], cell motility [13], adhesion [14], and cytokine production [15]. The transition of vascular smooth muscle cell (VSMC) phenotypes is facilitated by various angiogenic growth factors, the most extensively researched of which are transforming growth factor (TGF) β1 [16] and platelet-derived growth factor type BB (PDGF-BB) [17]. Unfortunately, due to material and ethical constraints, prior studies have focused mostly on histological evidence of spiral artery remodeling [18, 19]. Although the connection between insufficient remodeling of spiral arteries and obstetric complications has been known for more than four decades, our understanding of the cellular and molecular factors governing the intricate steps of this process is limited. While some headway has been achieved in recent years, further investigations are essential to gain a comprehensive understanding before viable therapeutic interventions can be developed. Here, we used single-cell RNA sequencing (scRNA-seq) of placental tissue to explore the mechanism of action between EVTs and dVSMCs in spiral artery remodeling.

Our study utilized single-cell RNA sequencing (scRNA-seq) to profile placental tissues from patients with very early-onset preeclampsia (EOPE, terminated before 28 weeks) and compared them with those of matched controls. Interestingly, the identification of differentially expressed genes (DEGs) in the EVTs via scRNA-seq revealed the involvement of Disabled-2 (DAB2) in cell communication, adhesion, and multicellular processes. DAB2, which has polypyrimidine tract-binding (PTB) domains and a proline-rich domain (PRD), plays a role in growth factor signaling, cytoskeletal reorganization, and cellular adhesion [20–22]. Originally identified in ovarian tumors [23], DAB2 has also been detected in various other tumor types [24–26]. Moreover, studies indicate that chemokines and their receptors play crucial roles in SpA regulation surrounding EVTs and the maternal–fetal microenvironment [27]. The regulation of trophoblast invasion and differentiation by the chemokine-CCR1 system is considered a key molecular mechanism of maternal vascular remodeling during early pregnancy in humans [28]. However, few reports have investigated whether the chemokine system is involved in the reprogramming of vascular smooth muscle cells and the vascular remodeling process. We investigated the influence of DAB2 on trophoblast function, its potential effects on VSMCs, and the underlying regulatory mechanisms involved.

## Materials and methods

This study was approved by the ethics review board of the Third Affiliated Hospital of Guangzhou Medical University. A detailed Materials and Methods section is available in the Supplemental Materials and Methods.

## Results

### Molecular analysis of EOPE and control placentas via single-cell RNA sequencing

**Fig. 1:**
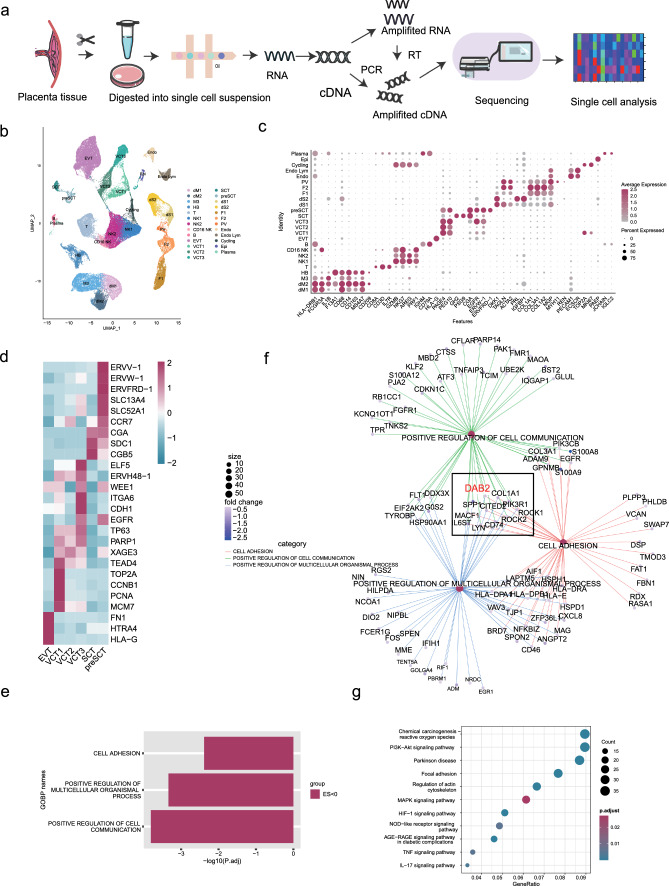
Single-Cell RNA Sequencing Analysis of EOPE Compared with Matched Control Gestation. **a** A schematic representation of the single-cell RNA sequencing analysis conducted on the placenta from EOPE and its corresponding control gestation. **b** A Uniform Manifold Approximation Plot (UMAP) showcasing individual cells, each distinguished by its specific cell type through unique coloring. **c** Dot plots illustrating the expression levels of recognized lineage markers in conjunction with co-expressed lineage-specific genes. **d** A heatmap that delineates the marker genes present across different trophoblast categories, encompassing EVT, VCT1s, VCT2, VCT3, SCT, and preSCT. **e** Go-biologicalanalysis of DEGs-downregulated in EVTs picked up the cell communication, multicellular organismal, and cell adhesion three biological processes. **f** The major enriched genes in three biological signaling functions in Fig. 1e. DAB2 was picked up for further research. **g** The enriched KEGG pathways of the DEGs- downregulated in VSMCs of EOPE compared with matched control gestation

To elucidate the molecular shifts at the maternal–fetal interface during midgestation in EOPE, we conducted scRNA-seq on placental tissues using the 10X Genomics platform. (Fig. 1a). After stringent quality control and doublet removal, we divided 27,330 high-quality cells into 25 unique subsets and visualized them via uniform manifold approximation and projection (UMAP) (Fig. 1b). The cell subsets of trophoblasts (including villous cytotrophoblasts [VCT1, VCT2 and VCT3]; presyncytiotrophoblasts [preSCTs]; syncytiotrophoblasts [SCTs]; extravillous trophoblasts [EVTs]; macrophages [dM1, dM2, and M3]; fetal macrophages [Hofbauer cells; HBs]; T cells; B cells; endothelial cells [Endo, Endo Lym]; epithelial cells [Epis]; cycling cells [Cyclings]; plasma cells [NK1, NK2, and CD16 NK]; decidual stromal cells [dS1 and dS2]; perivascular cells [PVs, classified in our study based on markers of vascular smooth muscle cells]; and fibroblasts [F1 and F2]) were identified based on the expression of well-defined marker genes (Fig. 1c, d).

Interestingly, Gene Ontology (GO) analysis of the DEGs downregulated in EVTs revealed three biological processes, cell communication, multicellular organization, and cell adhesion, which are related to the function of EVTs in the maternal–fetal interface (Fig. 1e). After assessing the related gene functions, we focused on DAB2, which was previously described as particularly intriguing (Fig. 1f). Moreover, Kyoto encyclopedia of genes and genomes (KEGG) analysis revealed enrichment of the PI3K-AKT pathway, MAPK pathway, hypoxia-inducible factor-1 (HIF-1) signaling pathway and other pathways in the VSMCs compared with those in the control group (Fig. 1g). This process involves the isolation of vascular smooth muscle cells (VSMC), degradation of the extracellular matrix (ECM) within the vascular wall, expansion of the blood vessel, transformation of the VSMC phenotype, migration away from the vascular wall, and subsequent apoptosis, leading to eventual removal by phagocytic macrophages [29]. Kaimoto et al. reported that the phosphatidylinositol 3-kinase/Akt signaling pathway was participated in regulating the dedifferentiation of VSMCs [30]. A study reported that PDGF-BB could increased rat aortic smooth muscle cells migration through the MAPK pathway shn [31]. Moreover, HIF-1 plays an important role in stress-responsive gene expression and is related to a number of stress factors, including metabolic stress, growth factors, and molecules present in the extracellular matrix (ECM) [32]. And KEGG pathway showed that might involved in spiral artery remodeling.

### DAB2 localization and expression in villous and decidua basalis tissues in early pregnancy

**Fig. 2:**
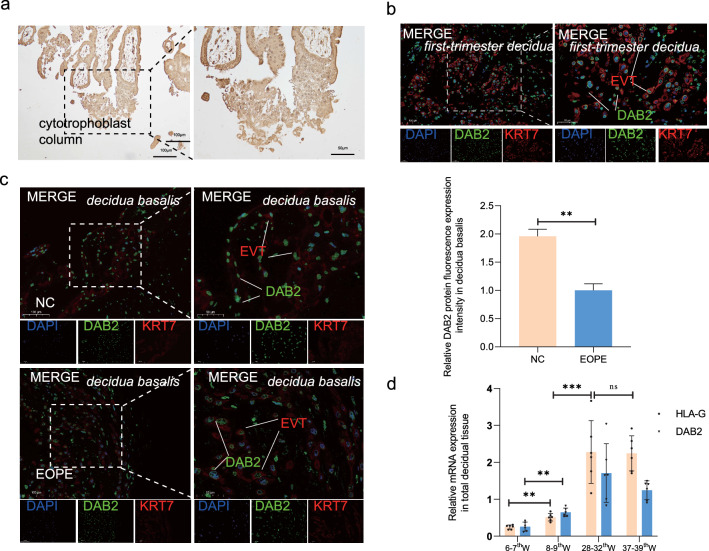
Localization and Expression of DAB2 in First-Trimester Villi, Early-Onset Preeclamptic, and Control Group decidua basalis. **a** DAB2 is localized in the cytrophoblast column of first-trimester villi. The brown signal denotes a positive DAB2 expression at this location (bar = 100 μm, 50 μm), n = 6. **b** KRT7 marks the extravillous trophoblast cells in decidua. DAB2, indicated by the green fluorescence, is localized within the cytoplasm of extravillous trophoblast cells in first-trimester decidua (bar = 100 μm, 50 μm), n = 6. **c** Immunofluorescence (IF) was utilized to compare the DAB2 expression between EOPE and gestational-matched normal pregnant decidua basalis. The results revealed a weaker fluorescence intensity in the EOPE group compared to the control group (NC *vs* EOPE: 1.734 ± 0.27 *vs* 1.064 ± 0.26, p** < 0.01). This suggests a down-regulation of DAB2 expression in the EVT of the EOPE group (bar = 100 μm, 50 μm), n = 6. **d** qRT-PCR analysis of DAB2 and HLA-G mRNA expression in lysates from decidua basalis tissues from 6–7th week, 8–9th week, 28–32th week to 37–39th week, n = 6. Above all each bar represents the mean ± SD, n = 6

Using immunohistochemistry and immunofluorescence, we evaluated the presence of DAB2 in early pregnancy tissues. DAB2 was abundantly expressed in various trophoblasts and was present in decidual extravillous trophoblasts (the green fluorescence indicates DAB2 and KRT7, and the red fluorescence represents EVT + cells in the decidua; Fig. 2a, b). KRT7 + cells were EVT + , and the results showed that DAB2 + cells had greater fluorescence intensity in the NC group than in the EOPE group (Fig. 2c, p** < 0.01). The transcript levels of DAB2 correlated with increasing levels of human leukocyte antigen G (HLA-G) in lysates from decidual basalis tissues collected during the first, middle and late trimesters (Fig. 1d). DAB2 expression increased from the first trimester to the second trimester but remained unchanged in the third trimester (the sample information is shown in Supplementary Table S2d). During the transition from the first to the second trimester of pregnancy, important events involving vascular remodeling occur, involving a range of molecular functions.

### DAB2 has a positive effect on HTR-8/SVneo cell survival and migration

**Fig. 3:**
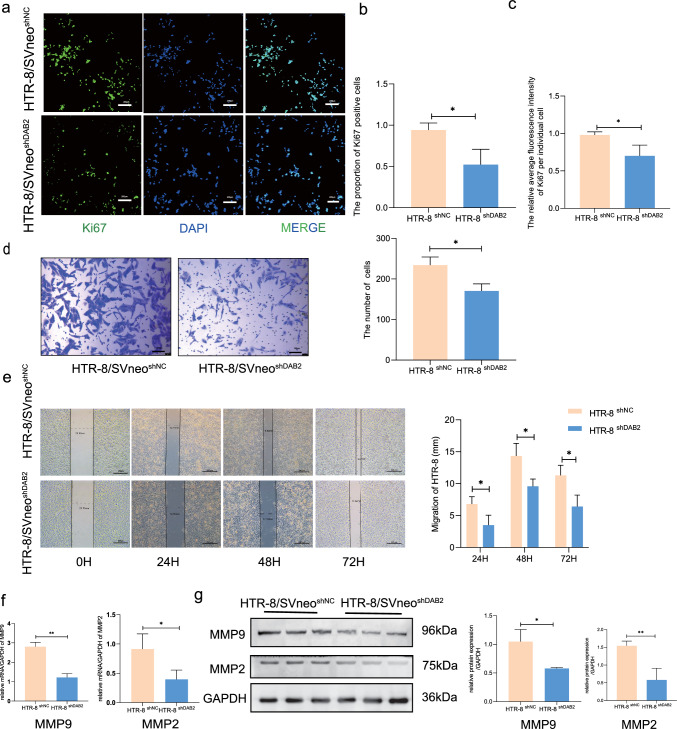
Knockdown of DAB2 Impedes Survival and Migration Functions of HTR-8/SVneo Cells. **a** Ki67 staining assay was used to detect the survival of HTR-8/SVneo cells. The HTR-8/SVneo^shNC^ group exhibited denser green fluorescence than the HTR-8/SVneo shDAB2 group, indicating that DAB2 knockdown diminishes HTR-8/SVneo proliferation. **b** The proportion of Ki67-positive HTR-8/SVneo cells (HTR-8/SVneo ^shNC^ *vs* HTR-8/SVneo ^shDAB2^: 0.94 ± 0.09 *vs* 0.52 ± 0.19, p* < 0.05), n = 3. **c** The relative average fluorenscence intensity of Ki67 per individual HTR-8/SVneo cells (HTR-8/SVneo ^shNC^ *vs* HTR-8/SVneo ^shDAB2^: 0.98 ± 0.04 *vs* 0.70 ± 0.15), n = 3. **d** Transwell assay wasused to detected the cell migration. HTR-8/SVneo^shNC^ group displayed a higher count of migrated cells than HTR-8/SVneo^shDAB2^ group (HTR-8/SVneo ^shNC^ *vs* HTR-8/SVneo ^shDAB2^: 234 ± 20 *vs* 170.3 ± 17.56, bar = 200 μm, p^*^ < 0.05). **e** The scratch assay was performed to track cell migration over intervals of 24, 48, and 72 h. HTR-8/SVneo^shNC^ group consistently exhibited greater migration distances than HTR-8/SVneo^shDAB2^ group in 24, 48 and 72 h, suggesting DAB2's role in enhancing HTR-8/SVneo cell migration (HTR-8/SVneo^shNC^ *vs* HTR-8/SVneo^shDAB2^: 24H: 5.117 ± 1.355 *vs* 3.947 ± 0.684; 48H: 9.58 ± 1.012 *vs* 6.3 ± 1.72; 72H: 13.037 ± 2.16 *vs* 9.86 ± 0.931, bar = 200 μm, all p^**^ < 0.01). **f** qRT-PCR showed that the mRNA level of MMP2 and MMP9 expression were decreased in HTR-8/SVneo^shDAB2^ group (mRNA level: HTR-8/SVneo^shNC^ *vs* HTR-8/SVneo^shDAB2^: MMP2: 0.91 ± 0.3 *vs* 0.4 ± 0.16, p^*^ < 0.05; MMP9: 2.80 ± 0.23 *vs* 1.22 ± 0.20, p** < 0.01). **g** Western blotting (WB) showed that the protein level of MMP2 and MMP9 expression were decreased in HTR-8/SVneo^shDAB2^ group (protein level: HTR-8/SVneo^shNC^ *vs* HTR-8/SVneo^shDAB2^: MMP2: 1.55 ± 0.13 *vs* 0.4 ± 0.16, p^*^ < 0.05; MMP9: 1.05 ± 0.21 *vs* 0.58 ± 0.02, p** < 0.01)

Given the diminished DAB2 expression in EVTs in EOPE decidual basalis, we investigated its impact on trophoblast cell behavior using the HTR-8/SVneo cell line. DAB2 was expressed in the HTR-8/SVneo cell cytoplasm (Fig. S1a), and then, we transfected HTR-8/SVneo cells with siRNAs (GenePharma, Suzhou, China), especially those targeting DAB2. The transfection efficiency was confirmed by quantitative real-time polymerase chain reaction (qRT–PCR) (Fig. S1b) and western blotting (WB) (Fig. S1c). Furthermore, HTR-8/SVneo cells were transfected with lentivirus (shRNA, siRNA#1) containing mCherry (Fig. S1d), and the transfection efficiency was also measured by qRT‒PCR and WB (Fig. S1e). The Ki67 staining results showed that knockdown of DAB2 suppressed HTR-8/SVneo cell viability (Fig. 3a). Transwell assays also revealed that migration was impaired after DAB2 was downregulated (Fig. 3d). A scratch assay (Fig. 3e) showed that the migration distance of the HTR-8/SVneo^shDAB2^ cells was shorter than that of the HTR-8/SVneo^shNC^ cells at the 24 h, 48 h and 72 h time points [Fig. 3c, p** < 0.01 (24 h, 48 h, 72 h)]. Furthermore, the expression of the associated molecular markers matrix metalloproteinase 2 and 9 (MMP2 and MMP9; Fig. 3f, g) was decreased in the downregulated group, suggesting that DAB2 could enhance HTR-8/SVneo cell survival and migration, potentially influencing vascular modifications in the spiral artery.

### Influence of EVT on hVSMC dedifferentiation

The phenotypic transition of VSMCs in response to environmental changes has been documented [33]. Within 6–10 gestational weeks, human decidua basalis was subjected to immunofluorescence analysis for CD31 and α-SMA in adjacent sections to identify endothelial cells and dVSMCs, respectively (Fig. S2a). The distribution of VSMCs allowed the identification of SPAs at different stages: unremodeled (Un-Rem), early remodeling (Early-Rem), secondary remodeling (Sec-Rem), and fully remodeled (Fully-Rem) (Fig. S2a). At the Un–Rem stage, a subset of dVSMCs exhibited morphological changes, such as misalignment and a rounder shape, albeit at a relatively low frequency. During the Early-Rem stage, the multilayered VSMCs surrounding these SPAs notably appeared looser and more separated, with a substantial number displaying misalignment and a rounder morphology. As the SPAs progressed to the Sec-Rem stage, the vasculature gradually disintegrated, leading to thinner vessel walls. In the Fully-Rem vessels, α-SMA and CD31 staining was barely detectable.

To examine the impact of EVTs on SPA VSMCs, we used a human aorta VSMC (hVSMC) cell line as a model. The hVSMC were subjected to 48 h of treatment with conditional medium (48 h culture supernatant from HTR-8/SVneo cells) and complete culture medium (DMEM + 10% FBS). Morphologically, immunofluorescence staining for α-SMA indicated a notable reduction in the number of α-SMA-positive cells and an increase in the number of rounded cells, suggesting that, compared with those in complete culture medium, the hVSMCs treated with CM-HTR/8-SVneo-treated cells underwent a transition to a synthetic phenotype (Fig. S3a). Additionally, qRT‒PCR and western blotting demonstrated significantly reduced expression levels of contractile markers (α-SMA, SM22α, calponin, and MYH-11) and protein levels (α-SMA and MYH11) in CM-HTR-8/SVneo-treated cells (Fig. S3b, c, p** < 0.01). According to prior research, VSMCs with a synthetic phenotype tend to migrate away from blood vessels and undergo apoptosis [34]. The protein levels of MMP2 and MMP9 were elevated in the HTR-8/SVneo culture medium treatment group (Fig. S3e, p** < 0.01 (MMP9), p* < 0.05 (MMP2)). A greater proportion of cells underwent apoptosis in the HTR-8/SVneo cell supernatant than in the control supernatant (Fig. S3f). Furthermore, 48-h supernatants from villous explants revealed decreased transcriptional levels of cell differentiation markers following 48 h of coculture with VSMCs, suggesting reduced cell differentiation in the CM-villous explant group (Fig. S3g, p** < 0.01, p* < 0.05). Based on our results, trophoblast cells can induce dedifferentiation in hVSMCs, promoting migratory and apoptotic processes that contribute to the remodeling of spiral arteries.

### DAB2 has a positive influence on HTR-8/SVneo-mediated hVSMC reprogramming

**Fig. 4:**
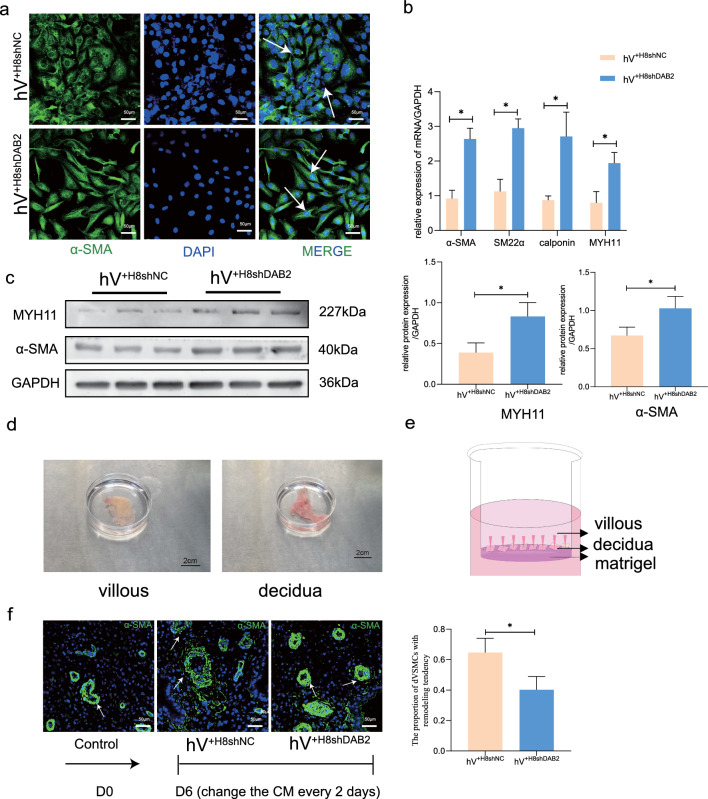
DAB2 in HTR-8/SVneo Facilitates hVSMCs Phenotypic Transition and Modulates SPA Remodeling. **a** Cell immunofluorescent staining of α-SMA in hVSMCs, which treated with the different group CM-HTR-8/SVneo (hV^+H8shNC^ group and hV^+H8shDAB2^ group), for investigate the effect of DAB2 differences in trophoblast cells on hVSMCs. The cells in hV^+H8shDAB2^ group remain hold the spindle shape, and the hV^+H8NC^ group turned to round shape, as the white arrows indicated. bar = 200 μm. **b** The relative mRNA level (calponin, MYH-11, α-SMA and SM22α) were detected by qRT-PCR. The contractile indicators in hV^+H8shNC^ group were lower than hV^+H8shDAB2^ group, which means that the DAB2 in trophoblasts could switch hVSMCs phenotype (hV^+H8shNC^ *vs* hV^+H8shDAB2^: calponin: 0.871 ± 0.122 *vs* 2.7 ± 0.702; MYH-11: 0.797 ± 0.324 *vs* 1.941 ± 0.306; α-SMA: 0.921 ± 0.236 *vs* 2.638 ± 0.309; SM22α: 1.125 ± 0.349 *vs* 2.947 ± 0.27, all p^*^ < 0.05). **c** The relative protein level (MYH-11, α-SMA) were detected by WB. The contractile indicators in CM of HTR-8/SVneo were lower than the hV^+H8shDAB2^ group (hV^+H8shNC^ *vs* hV^+H8shDAB2^: MYH-11: 0.389 ± 0.118 *vs* 0.8315 ± 0.098; α-SMA: 0.672 ± 0.11 *vs* 1.029 ± 0.157, all p^*^ < 0.05). **d** Images of the villi and decidua in the first trimester. **e** Schematic representation of the PDC model system. **f** Immunofluorescent staining of the dedicua tissue in co-cultured villous-decidua model with different conditioned media treatments. α-SMA-positive (green) represent the VSMC of spiral artery vascular. bar = 50 μm. Samples were were acquired from 12 pregnant women. The statistical analysis is performed based on the record from five cases for each section and five random views in each case. The proportions of remodeling are as follows: (hV^+H8shNC^ *vs* hV^+H8shDAB2^: 0.647 ± 0.09 *vs* 0.403 ± 0.087, p* < 0.05)

The positive influence of DAB2 on trophoblast survival and migration and the function of DAB2 in trophoblast cells on the vascular smooth muscle of the decidual spiral artery need further discussion. After 48 h, the culture media of HTR-8/SVneo^shDAB2^ and HTR-8/SVneo^shNC^ cells were collected and then added to hVSMCs (the hV^+H8shNC^ and hV^+H8shDAB2^ groups, respectively). The cells became round, which indicated that the hVSMCs in the hV^+H8shNC^ group might have adopted a synthetic phenotype compared with those in the hV^+H8shDAB2^ group (Fig. 4a, white arrow). The qRT‒PCR and western blotting results revealed that the transcription levels of contractional markers (Fig. 4b, p* < 0.05) and proteins (Fig. 4c, p* < 0.05) were significantly lower in the CM-HTR-8/SVneo^shNC^-treated cells than in the CM-HTR-8/SVneo^shDAB2^-treated cells.

A villous and decidua coculture system [35] was used to investigate the effect of conditioned medium-induced spiral artery remodeling (Fig. 4e). In the control group, decidual tissue without conditioned medium was collected, and the blood vessels exhibited complete morphology and no obvious signs of remodeling. Compared to those in the hV^+H8shNC^ group, the hV^+H8shDAB2^ group had fewer remodeled arteries, which were characterized by dVSMC layers (the green fluorescence represents a-SMA-positive cells) (Fig. 4f). dVSMC disruption and clear morphologic vascular remodeling changes, including disintegration of the dVSMC layers and a larger vascular lumen, were observed in the hV^+H8shNC^ group (white arrow). The proportion of vascular remodeling in each group was measured. We defined the phenomenon of vascular migration outside the vessel site as the remodeling group, and the other group was defined as the nonremodel group. The visual field where vascular remodeling occurred was selected, and five films were taken for quantitative analysis according to the number and proportion of vascular remodeling events. The results showed that the proportion of vascular remodeling was 64.7% in the hV^+H8shNC^ group and 40.3% in the hV^+H8shDAB2^ group, with a significant difference between the two groups (p* < 0.05).

Similarly, after 48 h of treatment, the supernatant of HTR-8/SVneo cells was collected from which DAB2 was knocked down, after which the 24-h migration ability was weaker than that of the NC group, and the number of apoptotic cells was less than that of the NC group (Fig. S4a, b, c).

### Elucidation of target genes and mediators influencing HTR-8/SVneo and hVSMC reprogramming

**Fig. 5:**
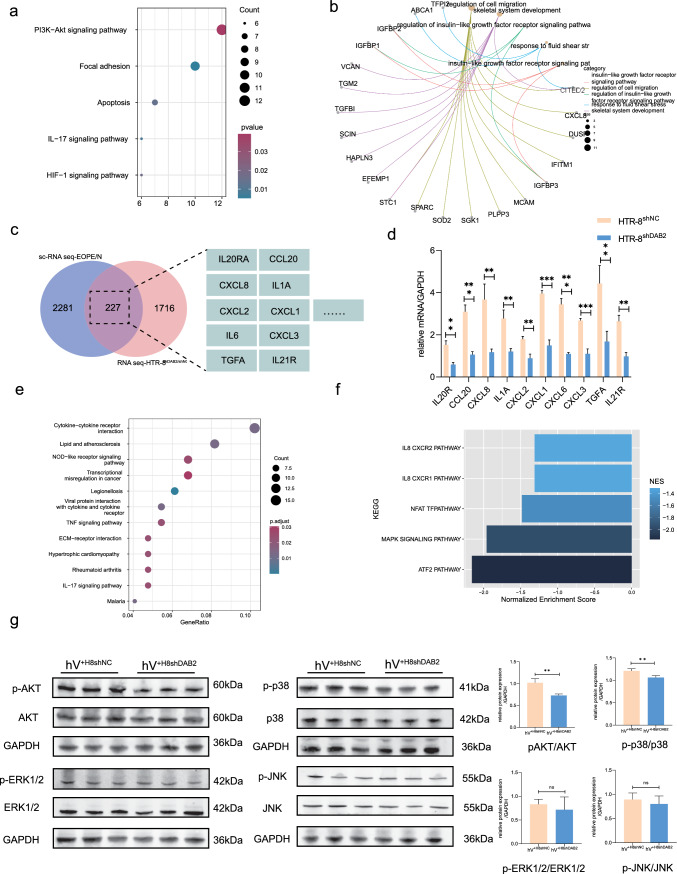
Comprehensive Discovery of Target Genes Modulating HTR-8/SVneo Function and the Mediators Governing hVSMCs Reprogramming. **a** KEGG enrichment pathway analysis of the intersection gene of down regulated genes in HTR-8/SVneo^shDAB2^ group of RNAseq and EVT genes in sc-RNA seq. **b** The enrichment circle figure showed that these co-down-regulated genes were enriched in insulin-like growth factor receptor signaling pathway and other pathways, which related with the cell survival and motility. **c** Intersection of downregulated genes from HTR-8/SVneo RNA-seq and EVT from scRNA-seq. Ten distinctly different chemokines and inflammatory factors potentially involved in the regulation of the maternal–fetal interface were selected for further enrichment analysis. **d** The mRNA level of chemokine and inflammatory factors were decreased in HTR-8/SVneoshDAB2 group then verified by qRT-PCR. ( HTR-8s^hNC^ *vs* HTR-8^shDAB2^: IL20R: 1.525 ± 0.193 *vs* 0.594 ± 0.093; CCL20: 3.442 ± 0.286; CXCL8: 3.670 ± 0.737 *vs* 1.177 ± 0.1521, IL1A: 2.771 ± 0.407 *vs* 1.207 ± 0.143, CXCL2: 1.795 ± 0.131 *vs* 0.889 ± 0.198; CXCL1: 3.098 ± 0.318 *vs* 1.062 ± 0.143; IL6: 3.951 ± 0.154 *vs* 1.498 ± 0.262 vs 1.099 ± 0.063; CXCL3: 2.676 ± 0.108 *vs* 1.105 ± 0.226; TGFA: 4.436 ± 0.853 *vs* 1.681 ± 0.486; IL21R: 2.641 ± 0.286 *vs* 0.977 ± 0.183, p* < 0.05, p** < 0.01). **e** Go biological analysis of co-downregulated genes in hV^+H8shDAB2^ group of hVSMCs RNA seq and PV of sc-RNA seq. **f** GSEA KEGG enrichment analysis of co-down-regulated genes in hV^+H8shDAB2^ group of hVSMCs RNA seq and PV of sc-RNA seq. The enrichment analysis of GSEA KEGG signaling pathway showed that IL8 CXCR1/2 signaling pathway, and MAPK signaling pathway were down-regulated by analyzing the differential gene expression levels of co-down-regulated genes in hV^+H8shDAB2^ group of hVSMC RNA seq and PV of sc-RNA seq. **g** After hVSMC was co-cultured with medium supernatants from different groups, WB was used to detect important molecules (AKT, p-AKT, p38, p-p38, p-ERK1/2, ERK1/2, p-JNK, JNK) in both pathways. The results showed that only PI3K/AKT pathway and p-p38 MAPK pathway were significantly different (hV^+H8shNC^ *vs* hV^+H8shDAB2^: p-AKT/AKT: 1.02 ± 0.09 *vs* 0.73 ± 0.03, p** < 0.01; p-p38/p38: 1.211 ± 0.05 *vs* 1.062 ± 0.04, p* < 0.05; p-ERK1/2/ERK1/2: 0.083 ± 0.11 *vs* 0.715 ± 0.27, p = 0.53; p-JNK/JNK: 0.893 ± 0.14 *vs* 0.80 ± 0.17, p = 0.49; n = 3)

We conducted an RNA-seq transcriptomic analysis of HTR-8/SVneo cells and hVSMC to discern the transcriptional changes in trophoblast cells after DAB2 knockdown and to pinpoint the mediators responsible for hVSMC reprogramming. A comparison revealed 2199 downregulated genes and 3317 upregulated genes that were significantly altered > 1.5-fold in the HTR-8/SVneo^shDAB2^ population compared with the HTR-8/SVneo^shNC^ population (p value < 0.05; Fig. S5a). We overlapped the DEGs identified via RNA-seq with the EVT DEGs from single-cell sequence data to obtain 227 genes that were involved in the PI3K-AKT pathway and the IL-17 signaling pathway, which are involved in cell migration and cell survival (Fig. 5a). Most of the pathways were associated with cell migration regulation and the insulin-like growth factor receptor signaling pathway, which could explain why the function of HTR-8/SVneo cells changed after DAB2 was knocked down (Fig. 5b). Interestingly, chemokines, known for recruiting leukocytes, are highly expressed at the maternal–fetal interface. In addition to immune cells, trophoblast cells are also involved in chemokine regulation [27].

**Fig. 6:**
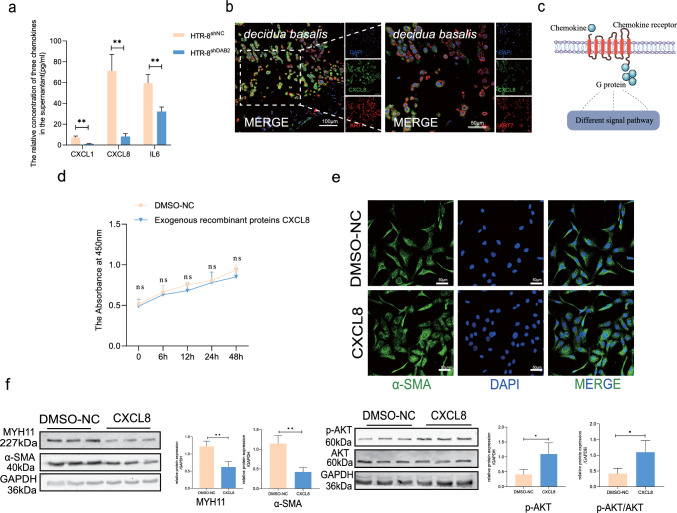
CXCL8 was expressed in trophoblast and could modulate hVSMCs dedifferentiation. **a** The relative concentration of CXCL1, CXCL8 and IL-6 in supernatant of HTR-8/SVneoshNC and HTR-8/SVneoshDAB2 were detected by Elisa analysis. The relative concentration expression of CXCL1, IL-6 and CXCL8 were exhibited. (HTR-8shNC *vs* HTR-8shDAB2: CXCL1: 7.313 ± 1.315 *vs* 1.164 ± 0.417, p** < 0.01; CXCL8: 71.29 ± 15.9 *vs* 8.164 ± 2.765, p** < 0.01; IL6: 59.49 ± 8.166 *vs* 32.19 ± 4.315, p** < 0.01, respectively). **b** CXCL8 (The green fluorensecen) was expressed in first trimester decidua basalis and located in EVTs (KRT7^+^ represents EVT cells in decidua). **c** Schematic representation illustrating the interaction of chemokines with seven-transmembrane G-protein-coupled receptors, leading to G-protein subunit dissociation, activation of signaling pathways, and subsequent cellular function modulation. **d** CCK8 cytotoxicity assay was performed at 6 h, 12 h, 24 h and 48 h time points, and there was no significant difference between them (DMSO-NC *vs* CXCL8: 0 h: 0.52 ± 0.06 *vs* 0.49 ± 0.05; 6 h: 0.67 ± 0.07 *vs* 0.65 ± 0.06; 12 h: 0.74 ± 0.04 *vs* 0.68 ± 0.06; 24 h: 0.78 ± 0.06 *vs* 0.79 ± 0.10; 48 h: 0.93 ± 0.05 *vs* 0.88 ± 0.06; p > 0.05). n = 6. **e** A morphological shift towards a rounded cell shape was observed after been treated with CXCL8, whereas cells in the DMSO control group maintained a spindle shape. **f** The dedifferentiation marker (MYH-11 and α-SMA) were examined by western-blot analysis. The protein expression of MYH-11 and α-SMA were decreased (DMSO-NC *vs* CXCL8: MYH-11: 1.222 ± 0.151 *vs* 0.619 ± 0.153, p^**^ < 0.01; α-SMA: 1.145 ± 0.203 *vs* 0.424 ± 0.111, p^**^ < 0.01) when treated with exogenous recombinant protein CXCL8, which means that CXCL8 could promote hVSMCs dedifferentiation and might participate in the process of spiral artery remodeling. AKT and pAKT were important indexes in the PI3K/AKT pathway. After treatment with CXCL8, the pAKT expression was increased and indicated that CXCL8 could activate PI3K signal pathway (DMSO-NC *vs* CXCL8: p-AKT: 0.4129 ± 0.168 *vs* 1.095 ± 0.379, p^*^ < 0.05; p-AKT/AKT: 0.4126 ± 0.167 *vs* 1.096 ± 0.379, p^*^ < 0.05)

We selected the top ten differentially expressed chemokines and inflammatory factors and verified the mRNA levels through qRT‒PCR (Fig. 5d). We also reviewed the relevant literature to identify the genes related to cell migration and various ontologies related to signaling and some vascular processes. Finally, the feasible target genes were identified as CXCL8, CXCL1, and IL6 because of their differential expression and relative expression. The concentration in the supernatant was subsequently verified via ELISAs (Elisa Kit, ABclonal, Wuhan, China). Compared with CXCL1 and IL-6, CXCL8 exhibited the most significant difference in expression between the two groups of supernatants (Fig. 6a). CXCL8 was selected as a mediator that might mediate dVSMC reprogramming.

For hVSMC, we analyzed the transcriptional shifts triggered by trophoblast 48-h HTR-8/SVneo-conditioned medium, and there were significant changes in gene expression. Specifically, in the hV^+H8shDAB2^ group, 398 genes were significantly suppressed, whereas 154 genes were upregulated with 1.5-fold changes (*p value < 0.05; Fig. S5c). After the DEGs identified from the hVSMC RNA sequencing and PV scRNA-seq data were overlapped, KEGG analysis revealed enrichment of genes related to cytokine‒cytokine receptor interactions, the ECM-receptor pathway, and the NOD-like receptor signaling pathway, which are related to the tissue remodeling process (Fig. 5e). GSEA KEGG analysis revealed that the IL8/CXCR1/2 pathway was suppressed in the EOPE group (Fig. 5f). According to the enrichment analysis of downregulated DEGs in EOPE in PV, the main enriched pathways included those involved in the regulation of cell differentiation, response to cytokines and apoptotic processes (Fig. S5e, f), which was also consistent with the hypothesis that in PE, spiral artery remodeling was impaired, smooth muscle cells were poorly responsive to chemokines, the dedifferentiation process was inhibited, and the dVSMC apoptotic process was reduced.

By reviewing past research on the pathways involved in migration and phenotypic transition, we speculated that the mitogen-activated protein kinase (MAPK) [31] and PI3K/AKT [30, 36, 37] signaling pathways may play a role in mediating downstream regulatory effects. In conclusion, AKT/p-AKT, ERK/p-AKT, JNK/p-JNK, and p38/p-p38 were assessed to investigate the effects of trophoblasts on hVSMC functions (Fig. 5g). The results showed that both the PI3K/AKT and MAPK/P38 pathways were promoted after treatment of smooth muscle cells with different supernatants, but the difference in the PI3K pathway was more significant (Fig. 5g, ** p < 0.01, *p < 0.05). In the next work, we examined the role of the PI3K pathway in mediating the dedifferentiation of hVSMCs.

### DAB2 through CXCL8/PI3K/AKT pathway regulate hVSMC dedifferentiation

Chemokines, which are particularly vital at the maternal–fetal interface, bind to G protein-coupled receptors, subsequently activating specific signaling pathways (Fig. 6c) [27]. The results showed that EVT cells strongly expressed CXCL8 (Fig. 6b). Exogenous recombinant CXCL8 protein (Abcam, ab259397, USA) was used to investigate the function of CXCL8. CXCL8 (10 ng/ml, according to the manual) was added to the cell culture medium for coculture for 48 h. According to the cellular immunofluorescence results, the cells changed from long fusiform to round, and the levels of MYH11 and a-SMA decreased, which indicated that CXCL8 promoted the dedifferentiation of hVSMCs (Fig. 6d, e). Furthermore, the results indicated that CXCL8 promoted hVSMC migration and apoptosis (Fig. S4d, e, f). Furthermore, CXCL8 activated the PI3K pathway, and the pAKT/AKT ratio was increased (Fig. 6f).

CXCL8 signaling plays a role mainly through two cell-surface receptors, CXCR1 and CXCR2 [38]. Navarixin (20 nM, CXCR1 and CXCR2 receptor antagonist, MCE, HY-10198, China) and a PI3K pathway inhibitor (LY29400, Selleck, S1105, USA) were used to inhibit the function of CXCL8 and the PI3K pathway to further determine whether hVSMC differentiation was due to the regulation of CXCL8 and the PI3K/AKT pathway. The cells were divided into the following groups: CM-HTR-8/SVneo^shNC^ + CXCR1/2 antagonist/LY29400, CM-HTR-8/SVneo^shNC^ + DMSO, CM-HTR-8/SVneo^shDAB2^ + CXCR1/2 antagonist/LY29400 and CM-HTR-8/SVneo^shDAB2^ + DMSO.

**Fig. 7:**
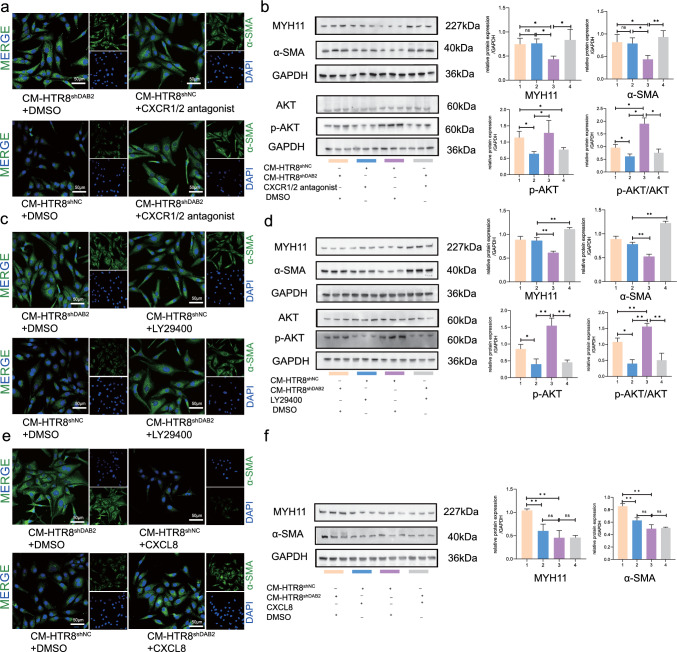
DAB2 in EVTs mediated CXCL8/PI3K/AKT pathway regulating hVSMC dedifferentiation. **a** Cell immunofluorescent staining of α-SMA in hVSMCs, which treated with the above four different supernatant to investigate the hVSMCs morphology four find out the effect on hVSMC dedifferentiation. CXCR1/2 antagonist could inhibit the function of CXCL8. When treated with the CXCR1/2 antagonist, hVSMC morphology remain spindle in CM-HTR/8-SVneo^shNC^ and CM-HTR/8-SVneo^shDAB2^ group. The CM-HTR/8-SVneo^shNC^ + DMSO group hVSMC turn to round and consistent with the previous results. At the same time, the CM-HTR/8-SVneo^shDAB2^ + DMSO group hVSMC remain spindle shape. **b** The relative protein level (MYH-11, α-SMA) were detected by WB. The contractile indicators were not downregulated after co-cultured with CXCR1/2 antagonist. The CM-HTR/8-SVneo^shNC^ + DMSO group find that could promote hVSMC dedifferentiation (CM-HTR/8-SVneo^shDAB2^ + DMSO *vs* CM-HTR/8-SVneo^shNC^ + CXCR1/2 antagonist *vs* CM-HTR/8-SVneo^shNC^ + DMSO group *vs* CM-HTR-8/SVneo^shDAB2^ + CXCR1/2 antagonist: MYH-11: 1.011 ± 0.245 *vs* 0.924 ± 0.1422 *vs* 0.583 ± 0.054 *vs* 1.294 ± 0.029, p^*^ < 0.05; α-SMA: 1.162 ± 0.223 *vs* 0.983 ± 0.101 *vs* 0.611 ± 0.014 *vs* 1.291 ± 0.147, p^*^ < 0.05, p^**^ < 0.01). Moreover, AKT and pAKT two molecular in PI3K pathway were detected by WB analysis (CM-HTR/8-SVneo^shDAB2^ + DMSO *vs* CM-HTR/8-SVneo^shNC^ + CXCR1/2 antagonist *vs* CM-HTR/8-SVneo^shNC^ + DMSO group *vs* CM-HTR-8/SVneo^shDAB2^ + CXCR1/2 antagonist: p-AKT: 1.141 ± 0.192 *vs* 0.639 ± 0.068 *vs* 1.283 ± 0.383 *vs* 0.767 ± 0.064, p^*^ < 0.05; p-AKT/AKT: 0.959 ± 0.1278 *vs* 0.616 ± 0.094 *vs* 1.903 ± 0.230 *vs* 0.7575 ± 0.1468, p^*^ < 0.05). n = 3. **c** Cell immunofluorescent staining of α-SMA in hVSMCs, which treated with the above four different supernatant to investigate the hVSMC morphology four find out the effect on hVSMCs dedifferentiation. When treated with the LY29400, hVSMCs morphology remain spindle in CM-HTR/8-SVneo^shNC^ and CM-HTR/8-SVneo^shDAB2^ group. The CM-HTR/8-SVneo^shNC^ + DMSO group hVSMC turn to round and consistent with the previous results. At the same time, the CM-HTR/8-SVneo^shDAB2^ + DMSO group hVSMCs remain spindle. **d** The relative protein level (MYH-11, α-SMA) were detected by WB. The contractile indicators were not downregulated after co-cultured with LY29400 (CM-HTR/8-SVneo^shDAB2^ + DMSO *vs* CM-HTR/8-SVneo^shNC^ + LY29400 *vs* CM-HTR/8-SVneo^shNC^ + DMSO group *vs* CM-HTR-8/SVneo^shDAB2^ + LY29400: MYH-11: 0.890 ± 0.071 *vs* 0.870 ± 0.066 *vs* 0.616 ± 0.030 *vs* 1.11 ± 0.032, p^**^ < 0.01; α-SMA: 0.890 ± 0.061 *vs* 0.782 ± 0.041 *vs* 0.525 ± 0.049 *vs* 1.22 ± 0.040, p^**^ < 0.01). pAKT was decreased when treated with LY29400 (CM-HTR/8-SVneo^shDAB2^ + DMSO *vs* CM-HTR/8-SVneo^shNC^ + LY29400 *vs* CM-HTR/8-SVneo^shNC^ + DMSO group *vs* CM-HTR-8/SVneo^shDAB2^ + LY29400: p-AKT: 0.875 ± 0.094 *vs* 0.379 ± 0.139 *vs* 1.592 ± 0.344 *vs* 0.523 ± 0.034, p^*^ < 0.05, p^**^ < 0.01; p-AKT/AKT: 1.079 ± 0.1215 *vs* 0.399 ± 0.127 *vs* 1.560 ± 0.099 *vs* 0.508 ± 0.2168, p^*^ < 0.05). n = 3. **e** In order to further verify the critical role of CXCL8, exogenous factors of CXCL8 were added to different supernatants to observe whether CXCL8 could restore the dedifferentiated phenotype of the supernatant in the CM-HTR-8/SVneo^shDAB2^ group. The results showed that after CXCL8 was added to the supernatant in the knockout group, the cells turned round. The results suggest that CXCL8 is one of the key factors in dedifferentiation of hVSMC. **f** Consistent with the results of cell fluorescence, the differentiation index decreased after the addition of CXCL8 (CM-HTR/8-SVneo^shDAB2^ + DMSO *vs* CM-HTR/8-SVneo^shNC^ + CXCL8 *vs* CM-HTR/8-SVneo^shNC^ + DMSO group *vs* CM-HTR-8/SVneo^shDAB2^ + CXCL8: MYH-11: 1.044 ± 0.032 *vs* 0.603 ± 0.145 *vs* 0.457 ± 0.155*vs* 0.460 ± 0.047, p^**^ < 0.01; α-SMA: 0.858 ± 0.042 *vs* 0.629 ± 0.042 *vs* 0.493 ± 0.066 *vs* 0.508 ± 0.012, p^**^ < 0.01, ns means there was no difference between them). n = 3

After the CXCR1/2 antagonist was added to CM-HTR-8/SVneo^shNC^, the hVSMCs maintained the shape of a long spindle, and the dedifferentiation process was inhibited (Fig. 7a). Moreover, there were no significant differences among the other three groups. After the CXCR1/2 antagonist was added to the CM-HTR-8/SVneo^shNC^ group, more contractile markers were expressed than in the DMSO group (Fig. 7b). These results indicated that the CXCL8 in the supernatant might have a critical effect in the dedifferentiation process. Moreover, after CM-HTR-8/SVneo cells were treated with the CXCR1/2 antagonist, the expression of pAKT was reduced compared with that in the DMSO group. The other trend was consistent with the above result.

After incubation with LY29400, the cells in the CM-HTR-8/SVneo^shNC^ group remained spindle-shaped. The cells in the CM-HTR-8/SVneo^shNC^ + DMSO group were round (Fig. 7c). Similarly, contractile markers were detected by WB. After LY29400 was added to CM-HTR-8/SVneo^shNC^ cells, the expression of MYH11 and α-SMA was greater than that in the DMSO group (Fig. 7d). Moreover, pAKT was downregulated, but there was no significant difference between the two groups when LY29400 cells were cocultured with CM-HTR-8/SVneo^shNC^ or CM-HTR-8/SVneo^shDAB2^.

To further verify whether CXCL8 is a mediator of dedifferentiation in hVSMC, we added exogenous CXCL8 protein to different subgroup supernatants, and cell morphological changes and the expression of differentiation factors were detected to determine whether CXCL8 could rescue the process of dedifferentiation. The results showed that the cells became round after the addition of CXCL8 to the knockdown group (Fig. 7e). The results showed that CXCL8 secreted by trophoblasts was an important key factor in regulating hVSMC dedifferentiation.

### Preliminary investigation of the mechanism of DAB2-mediated regulation of CXCL8

The HTR-8/SVneo RNA-seq analysis showed that the mRNA levels of CXCL8 were decreased after DAB2 was knocked down. To determine how DAB2 modulates CXCL8, we employed protein mass spectrometry (MS) to identify DAB2-interacting proteins. After excluding the influence of negative controls, we identified a total of 294 proteins interacting with DAB2. GO and KEGG enrichment analyses of the molecules revealed that the signaling pathways were mainly involved in cell migration and differentiation and in the survival and migration of trophoblast cells (Fig. S6a, b). After overlapping the RNA-seq and IP-MS data, we identified Rho guanine nucleotide exchange factor 2 (GEF-H1 or ARHGEF2) (Fig. S6c, d). GEF-H1 promoted the conversion of GDP to GTP and subsequently regulated Rho-GTPases. Rho GTPases are a family of small G proteins that play major roles in cell migration and the metastatic process and participate in cellular processes that regulate the cytoskeleton [39]. The cells were divided into the input group (the HTR-8/SVneo cell protein sample), the IP-DAB2 group (the sample in which proteins that interact with DAB2 were pulled down according to the IP kit), and the IgG negative control group. The interaction of DAB2 with GEF-H1 was verified by co-IP analysis (Fig. S6g), and the GEF-H1 protein level was found to be decreased in EOPE placental tissues (Fig. S6h). Moreover, we used Genome Browser (https://genome.ucsc.edu/) and PROME (https://alggen.lsi.upc.es/cgi-bin/promo_v3) analyses to predict the binding factors of CXCL8, and a total of 162 transcription factors were predicted (Fig. S6e). Among the three transcription factors, NF-kB was decreased after DAB2 was knocked down in HTR/8-SVneo cells (Fig. S6h). NF-κB is a vital mediator of placentation and can regulate placentation through the modification of cytokine expression, as well as cell phenotype and function [40]. We hypothesized that DAB2 might interact with GEF-H1 to activate the Rho/NF-kB pathway to regulate CXCL8 expression, which needs further validation.

## Discussion

The clinical course of EOPE is highly unpredictable, and most commonly used criteria for severity, such as the magnitude of hypertension and proteinuria, are poorly associated with maternal and fetal outcomes [41, 42]. This uncertainty complicates decisions regarding the optimal timing for delivery, underscoring the need for more reliable biomarkers or molecular targets. Our study contributes to this field by revealing the potential role of Disabled-2 (DAB2) in the pathophysiology of EOPE. Remarkably, we showed that DAB2 may regulate the secretion of C-X-C motif chemokine ligand 8 (CXCL8), which subsequently activates the phosphoinositide 3-kinase/protein kinase B (PI3K/AKT) pathway in hVSMCs, as shown in Graphical abstract. This pathway is crucial for spiral artery remodeling, a key process in placentation. Our findings not only illuminate a novel aspect of DAB2 function but also hint at its broader implications in vascular biology and preeclampsia management.

Importantly, in this study, we elucidated the role of DAB2 in modulating the function and interaction of trophoblasts and vascular smooth muscle cells (VSMCs) during spiral artery remodeling. Several studies have reported the immunomodulatory effect of DAB2, but few studies have investigated the function of trophoblast. With its multiple binding domains, DAB2 has emerged as a key orchestrator in various signaling pathways, facilitating complex protein‒protein interactions [43]. Intriguingly, parallels drawn from oncological studies in which DAB2 expression was associated with tumor progression suggest that DAB2 may play a unique role in promoting cell viability and migration [44]. These cancer research findings are consistent with our observations, suggesting that DAB2 enhances EVT’s ability to modify vascular structures, akin to facilitating tumor cell migration and invasion [45, 46]. When DAB2 was knockdown, we found that the dedifferentiation process of hVSMC was blocked, suggesting that DAB2 might be involved in the regulation between EVTs and dVSMCs. We validated the importance of DAB2 for VSMC dedifferentiation via placental tissue and cell models in vitro. At the maternal–fetal interface, chemokines, such as CXCL14, CXCL12, CCL24 and so on have been verified expressed in primary trophoblast, might regulate intercellular communication [47, 48]. Of particular interest is CXCL8, which is known to be involved in inflammation-related diseases. Ruhul H Choudhury.et al. reported that the CXCL8 was secreted by EVTs and then induce endothelial cell expressed CCL14 and CXCL6 [49]. In addition, CXCL8 can induce immune cells to participate in the occurrence of vascular remodeling [50]. Moreover, CXCL8 can promote trophoblast cells migration and promote the progression of spiral artery remodeling [51]. Therefore, we investigated whether EVT-derived CXCL8 is involved in the regulation of decidual vascular smooth muscle cell dedifferentiation. After the function of CXCL8 was inhibited, the cells exhibited a long fusiform morphology, and the MYH11 and α-SMA levels did not decrease compared with those in the DMSO group. We found that there was no difference between the CM-HTR-8/SVneo^shDAB2^ and CM-HTR-8/SVneo^shNC^ added with the CXCR1/2 group, which indicates that CXCL8 plays an irreplaceable role in regulating hVSMC dedifferentiation and there are other factors that regulate the PI3K signaling pathway. Moreover, the addition of exogenous CXCL8 to the DAB2 knockdown group supernatant rescued the abnormal dedifferentiation of hVSMC. However, pAKT expression differed between the CM-HTR-8/SVneo^shDAB2^ group and the CM-HTR-8/SVneo^shNC^ with the CXCR1/2 group, indicating that other cytokines, except CXCL8, activate the PI3K pathway. A study on asthma revealed that activating the PI3K/AKT signaling pathway could promote the migration and proliferation of hVSMC [52]. Additionally, PDGF-ββ could activate phosphorylation of Akt and induce proliferation and migration of VSMCs [53]. When the PI3K inhibitor LY29400 was added to the supernatant of HTR-8/SVneo^shNC^ cells, the expression of p-AKT decreased, the cell morphology also remained long and fusiform, and the contractile indices did not decrease. It indicated that PI3K pathway plays an important role in hVSMC dedifferentiation and could broaden our understanding.

Furthermore, our IP-MS findings indicated an interaction between DAB2 and GEF-H1, as verified by co-IP analysis. GEF-H1 is implicated in several cellular processes, from barrier permeability to cancer [54]. Notably, Rho GTPases, which are pivotal for cell migration and cancer metastasis, may also influence the tumor microenvironment and inflammation [39]. NF-κB is a vital mediator of placentation [40] and a transcription factor of CXCL8 [55, 56]. These findings link DAB2's function to broader cellular processes such as cell migration and inflammation, further emphasizing its potential as a therapeutic target in managing disorders related to placental insufficiency.

While our study provides important insights into the role of DAB2 in spiral artery remodeling and its broader implications in EOPE, there are certain limitations, and further research is needed. One of the primary limitations is the limited size of our sequencing sample, which may affect the generalizability of our findings. In the control group, we selected samples from women who had preterm birth, which may introduce some bias. Additionally, the absence of spatiotemporal expression data for DAB2 limits our ability to precisely delineate its role throughout the different stages of EOPE. Understanding the temporal and spatial dynamics of DAB2 expression is crucial for fully comprehending its function in placental development and pathology. Moreover, our study predominantly relies on in vitro models. While these models are invaluable for dissecting molecular pathways and cellular interactions, they cannot replicate the complexity of in vivo systems. Therefore, there is a pressing need for comprehensive in vivo studies to confirm and extend our findings.

In conclusion, our study substantially enhances the understanding of placental biology and EOPE, particularly highlighting the complex mechanisms underlying spiral artery remodeling. The role of DAB2 in this process, as evidenced by its influence on EVT and hVSMC interactions, opens new avenues for maternal–fetal medicine research. Future studies should focus on elucidating the comprehensive role of DAB2 in various aspects of EOPE pathology, including its impact on immune responses and its potential as a biomarker for early detection and prognosis. Additionally, translating these findings into clinical practice remains a challenge. Potential therapeutic strategies, such as targeted inhibition of DAB2-mediated pathways or modulation of chemokine signaling, could pave the way for novel treatments for EOPE. These approaches might also provide insights into the management of other placental disorders and complications related to impaired vascular remodeling.

### Supplementary Information

Below is the link to the electronic supplementary material.

Supplementary file1 (XLS 217 KB)

Supplementary file2 (DOCX 32 KB)

Supplementary file3 (PDF 396462 KB)

Supplementary file4 (DOCX 35 KB)

## Data Availability

The data supporting the findings of this study are found in the article and the supplementary material. The corresponding author will make all relevant raw data available upon reasonable request.
