# Supplementary material for: Identification of the role of DAB2 and CXCL8 in uterine spiral artery remodeling in early-onset preeclampsia
Source: Cell Mol Life Sci. 2024 Apr 13;81(1):180. doi: 10.1007/s00018-024-05212-4 (PMC11016014; doi:10.1007/s00018-024-05212-4)
Supplement: Supplementary file 2 — Supplementary file2 (DOCX 32 KB) [file 18_2024_5212_MOESM2_ESM.docx]

Figure S1. DAB2 knockdown in HTR-8/SVneo cells

a. HTR-8/SVneo cell line was used as an extra-trophoblast model. DAB2 was located in the cytoplasm (Green fluorescence) of HTR-8/SVneo cells (bar=200μm).

b. HTR-8/SVneo cells were transfected by three different sequence siRNA to knock down the target gene. qRT-PCR showed that DAB2 mRNA expression was significantly decreased in the sequence 1 (si ^1#^) compared with the other two sequence.

c. Next, WB showed that DAB2 protein expression was significantly decreased in the sequence 1 (si ^1#^) compared with the other two sequence (si ^2#^ and si ^3#^).

d. HTR-8/SVneo cells were transfected by LV^mCherry^ (Red fluorescence) with polybrene. HTR-8/SVneo cells were expressed red fluorescent, which represent Lentivirus were transfected in HTR-8/SVneo cells successfully (bar=200μm).

e. qRT-PCR and WB showed that the level of DAB2 mRNA and protein expression were significantly decreased (HTR-8/SVneo^shNC^ *vs* HTR-8/SVneo^shDAB2^: 0.92 ± 0.14 *vs* 0.24 ± 0.05, p^**^＜0.01).

Figure S2. The dynamic transformation of SPA–VSMCs along the process of SPA remodeling in early pregnant decidua.

a. Immunofluorescent staining of CD31 (red) and αSMA (green) in the adjacent sections to identify different SPA remodeling stages. As the stage progresses, SPA–VSMCs gradually separated and misaligned. The stage of un-remodeling showed the vessel was intact. The stage of early-remodeling showed that the walls of the blood vessels begin to loosen, and there are also individual smooth muscle cells showing up around the blood vessels. The stage of second-remodeling showed the vascular wall is incomplete, blood vessel walls gradually thin and the blood vessel morphology gradually disappeared. The last stage was fully-remodeling, that the original spiral artery completely disappeared, forming a larger vascular cavity. White arrows indicate rounded and migrated SPA–VSMCs.

Figure S3. Extravillous trophoblast could promote hVSMC migration and apoptosis.

a. Cell immunofluorescent staining of α-SMA in hVSMCs, which treated with the 48h cultured medium of HTR-8/SVneo (CM-HTR-8/SVneo) and the complete culture medium (CM-Control, DMEM + 10% FBS), for investigate the effect of trophoblast on hVSMCs. The result showed that the cell morphology turned to round from fusiform in the CM-HTR-8/Svneo group. White arrows indicate round-shaped cells. bar=50μm, n=3.

b. The relative mRNA level (calponin, MYH-11, α-SMA and SM22α) were detected by qRT-PCR. The contractile indicators in hV^+CM-HTR-8/SVneo^ group were lower than hV^+CM-Control^ group, which means that the trophoblasts might switch hVSMCs phenotype (hV^+CM-Control^ *vs* hV^+CM-HTR-8/SVneo^: calponin: 0.871 ± 0.122 *vs* 2.700 ± 0.702; MYH-11: 0.797 ± 0.324 *vs* 1.941 ± 0.306; α-SMA: 0.921 ± 0.236 *vs* 2.638 ± 0.309; SM22α: 1.125 ± 0.349 *vs* 2.947 ± 0.27, all p^*^＜0.05, n=3 ).

c. The relative contractile protein level (MYH-11, α-SMA) were detected by WB. The contractile indicators in CM of HTR-8/SVneo treatment group were lower than the control group (hV^+CM-Control^ *vs* hV^+CM-HTR-8/SVneo^: MYH-11: 0.797 ± 0.324 *vs* 1.941 ± 0.306; α-SMA: 0.921 ± 0.236 *vs* 2.638 ± 0.309, all p^*^＜0.05, n=3).

d. hVSMCs had more migrated cells when exposure in CM-HTR-8/SVneo than control group( hV^+CM-control^ *vs* hV^+CM-HTR-8/SVneo^: 95.33 ± 8.622 *vs* 142.7 ± 9.713, p^*^＜0.05, n=3).

e. The relative protein level of MMP2 and MMP9 were detected by WB. The expression of MMP2 and MMP9 were lower in hV^+CM-Control^ than hV^+CM-HTR-8/SVneo^ group (hV^+CM-Control^ *vs* hV^+CM-HTR-8/SVneo^: MMP2: 1.712 ± 0.31 *vs* 0.867 ± 0.14, p^*^＜0.05; MMP9: 2.15 ± 0.25 *vs* 0.96 ± 0.06, p^**^＜0.01).

f. hVSMC had more apoptotic cells when exposure in CM-HTR-8/SVneo than the control group (Proportion of apoptosis: hV^+CM-Control^ *vs* hV^+CM-HTR-8/SVneo^: 13.11 ± 2.75 *vs* 21.52 ± 2.81, p^*^＜0.05).

g. The first trimester decidua tissue at 6-8 weeks of gestation, took the tip part of the villi, placed it on matrigel for growth, collected the supernatant at 48 hours, and co-cultured with hVSMC for 48 hours. The mRNA levels of contractility markers showed that cell contraction markers were decreased after treatment with explant supernatant (mRNA level: hV^+Control^ *vs* hV^+CM-villous explants^: α-SMA: 3.55 ± 0.46 *vs* 1.87 ± 0.61, p^*^＜0.05; SM22α: 4.10 ± 1.10 *vs* 1.62 ± 0.60, p^*^＜0.05; calponin: 2.86 ± 0.09 *vs* 0.78 ± 0.06, p^**^＜0.01; MYH11: 3.98 ± 0.62 *vs* 1.03 ± 0.36, p^**^＜0.01).

Figure S4. DAB2 in HTR-8/SVneo Facilitates hVSMC reprogramming via CXCL8/PI3K pathway -regualted hVSMCs migration and apoptosis

a. hVSMC had less migrated cells when exposure in CM-HTR-8/Svneo^shDAB2^ than the CM-HTR-8/Svneo^shNC^ group (hV^+H8shNC^ *vs* hV^+H8shDAB2^: 198.9 ± 7.91 *vs* 128.9 ± 18.11, p**＜0.01, n=3).

b. The relative protein level of MMP2 and MMP9 were detected by WB. The expression of MMP2 and MMP9 were lower in hV^+H8shDAB2^ than hV^+H8shNC^ group (hV^+H8shNC^ *vs* hV^+H8shDAB2^: MMP2: 1.011 ± 0.21 *vs* 0.539 ± 0.07, p*＜0.05; MMP9: 0.927 ± 0.19 *vs* 0.45 ± 0.11 , p*＜0.05).

c. hVSMC had more apoptotic cells when exposure in CM-HTR-8/Svneo^shNC^ than the CM-HTR-8/Svneo^shDAB2^ (Proportion of apoptosis: hV^+H8shNC^ *vs* hV^+H8shDAB2^: 23.47 ± 1.78 *vs* 11.27 ± 1.03, p*＜0.05, n=3).

d. hVSMC had less migrated cells when exposure in CXCL8-co-culturer group than the DMSO-control group ( DMSO-NC *vs* CXCL8: 100.3 ± 7.57 *vs* 151.0 ± 16.37, p**＜0.01, n=3).

e. The relative protein level of MMP2 and MMP9 were detected by WB. The expression of MMP2 and MMP9 were increased in CXCL8-co-culturer group than the DMSO-control group (DMSO-NC *vs* CXCL8: MMP2: 0.723 ± 0.15 *vs* 1.35 ± 0.08, p*＜0.05; MMP9: 0.55 ± 0.06 *vs* 1.27 ± 0.25 , p**＜0.01, n=3).

f. hVSMC had more apoptotic cells when exposure in CM-HTR-8/Svneo^shNC^ than the CM-HTR-8/Svneo^shDAB2^ (Proportion of apoptosis: DMSO-NC *vs* CXCL8: 15.37 ± 0.38 *vs* 10.93 ± 0.78, p**＜0.01, n=3).

g. CXCR1/2 inhibitor was added to inhibit the function of CXCL8, and the migration ability was reduced. The migrated cells were exhibited in four groups: (CM-HTR/8-SVneo^shDAB2^ + DMSO *vs* CM-HTR/8-SVneo^shNC^ + CXCR1/2 antagonist *vs* CM-HTR/8-SVneo^shNC^ + DMSO group *vs* CM-HTR-8/SVneo^shDAB2^ + CXCR1/2 antagonist: 149.7 ± 15.28 *vs* 113.3 ± 7.10 *vs* 228.7 ± 32.53 *vs* 166.0 ± 11.53. p*＜0.05, p**＜0.01, n=3).

PI3K inhibitor was cocultured with the two different contioned medium, and the migration ability was reduced. The migrated cells were exhibited in four groups: (CM-HTR/8-SVneo^shDAB2^ + DMSO *vs* CM-HTR/8-SVneo^shNC^ + LY29400 *vs* CM-HTR/8-SVneo^shNC^ + DMSO group *vs* CM-HTR-8/SVneo^shDAB2^ + LY29400: 161.7 ± 4.93 *vs* 127 ± 11.14 *vs* 203.3 ± 18.50 *vs* 138.7 ± 8.15. p*＜0.05, p**＜0.01, n=3).

Figure S5. Comprehensive Discovery of Target Genes Modulating HTR-8/SVneo Function and the Mediators Governing hVSMC Reprogramming

a. The volcano plot of HTR-8/SVneo cells, showing 2199 down-regulated genes and 3317 up-regulated genes that were significantly altered >1.5 folds after DAB2 knockdown.

b. The GSEA (Gene Set EnrichmentAnalysis) GO-analysis of downregulated DEGs were mainly enriched in positive regulation of metabolic process, extracellular space and so on, which might relate to cell matrix regulation.

c. The volcano plot of hVSMCs after co-cultured with two different contioned medium. The knockdown cultured medium group significantly suppressed 398 genes and upregulated 154 genes according to 1.5 folds changes.

d. The KEGG pathway analysis classification of downregulated DEGs were mainly enriched in IL17 pathway, cytokine pathway and chemokine pathway, which were related with the hVSMCs repragraming process.

e. Circle plot showed that the enrichment analysis of down-regulated genes in PV of the EOPE group showed that the main enrichment pathways were apoptotic process, regulation of cell differentiation and respons to cytokines.

f. GSEA enrichment annalysis showed that apoptotic process, response to cytokines and cell differentiation were suppressed, which was also consistent with the theoretical PE situation.

g. Schematic representation of the PI3K pathway.

h. Schematic representation of the MAPK pathway

Figure S6. Exploration of the mechanism of DAB2 regulating CXCL8

a. GO analysis of protein mass spectrometry results.

b. KEGG analysis of protein mass spectrometry results.

c. Integration of mass spectrometry results with HTR-8/SVneo sequencing identified four potential molecules that might interact with DAB2

d. Based on prior literature, ARHGEF2 (also known as GEF-H1) plays a role in the Rho signaling pathway and could active its subsequent transcriptional factors.

e. Predicted transcription factors in the website intergated with the down-regulated gene of the RNA seq of HTR-8/SVneo in the knockdown group. As per previous research, the vital transcriptional regulatory factor of CXCL8 might be NFkB.

f. Heat map results of the seven targeted transcription factors showed that they were down-regulated in the HTR-8/SVneo^shDAB2^ group.

g. The interaction between GEFH1 and DAB2 was verified by ip assay. Notably, GEFH1was found to be downregulated in placental tissues of EOPE.
