# Supplementary material for: Identification of the role of DAB2 and CXCL8 in uterine spiral artery remodeling in early-onset preeclampsia
Source: Cell Mol Life Sci. 2024 Apr 13;81(1):180. doi: 10.1007/s00018-024-05212-4 (PMC11016014; doi:10.1007/s00018-024-05212-4)
Supplement: Supplementary file 4 — Supplementary file4 (DOCX 35 KB) [file 18_2024_5212_MOESM4_ESM.docx]

*Supplymentary Materials and Methods*

1. Patients Enrollment and Placental Tissue Collection

Human placenta and decidua samples were sourced from the Department of Obstetrics and Gynecology at the Third Affiliated Hospital of Guangzhou Medical University, with written consent from all participants. The cohort included patients with singleton-term pregnancies undergoing elective caesarean section and patients diagnosed with EOPE [1] based on the ISSHP 2018 criteria [2]. The control group was the preterm birth group matched for gestational age without other complications, labeled as Normal Control (NC) group hereafter enrolled in this study. The demographic characteristics of participants in the EOPE group and the normal group used for sc-RNA seq are displayed in supplymentry Table S1 and the sample information used for verification in the experimental section of this paper is shown in Supplementary Table S2.

1. Isolation of single-cell, single-cell sequencing and analysis

For scRNA-seq, cells from the placenta tissues were isolated by using the previously described method [3, 4]. Briefly, placenta tissues were minced, enzymatically digested for 30 min, treated with DMEM culture medium containing 10% FBS (corning, 35-030-CV) and filtered (70-μm nylon filter; 431751). Red blood cells and dead cells were removed by kit (Red Blood Cell Removal Solution, Beyotime, C3702; Dead Cell Removal Kit, Miltenyi Biotec, 130-090-101). Then, viable cells were used for scRNA-seq library construction using the Chromium Single Cell 3’ kit v2 (10X Genomics), following the manufacturer’s instructions. After quantifying the DNA libraries, the single-cell libraries’ sequencing was performed using the Illumina Platform (HiSeq X Ten System). The CellRanger (10X Genomics) analysis pipeline was used to generate a digital gene expression matrix from the sequencing data as previously described [5].

For placenta samples, sequencing data from the 4 EOPE patients and 3 NC group women with a comparable duration of gestational age were analyzed. Seurat v.3 was used for downstream analysis. The following criteria were then applied to each cell of all samples: gene number between 200 and 6000 and mitochondrial gene percentage proportion < 0.05. We utilized the R package scDblFinder [6](version 1.9, https://github.com/plger/scDblFinder) to eliminate doublets/multiplets in single-cell sequencing data according to the previous reports[7, 8]. After filtering, a total of 27330 cells were left for the following analysis.

1. EVT differential gene expression analysis, gene functional annotation

Wilcox in Seurat v.3 (FindAllMarkers function) was used to perform differential gene expression analysis. For each cluster of placenta cells, DEGs of EOPE placenta were generated to compare with the same cell type of the NC group. We caught our eyes on the DEGs of EVT in this study and EVT GO analysis was performed

1. HTR-8/SVneo cells and hVSMCs Culture

Human trophoblast cell lines HTR-8/SVneo and the T/G HA-VSMC cell line (CRL-1999) were gifts from my former colleagues Dr. Yang Zhang [9] and Dr. Mengying Wu [10], respectively. Cells were cultured in DMEM medium (Gibco, USA) supplemented with 10% fetal bovine serum (FBS; Corning, USA). Cells were maintained in a humidified incubator (5% CO2 and 95% air, Thermo Scientific, USA) at 37°C.

1. HTR-8/SVneo cells transfection

To investigate the function of DAB2 in HTR-8/SVneo, we transfected three si-DAB2 in HTR-8/SVneo. The three primers of si-DAB2 which were purchased in GenePharma (Suzhou, China) were as follows: si-DAB2^#1^: sense: GUGAGGCCCUAAUGAUUCUTT; antisense: AGAAUCAUUAGGGCCUCACTT: si-DAB2^#2^: sense: GACACCUUCUUCGUUUGAUTT; antisense: AUCAAACGAAGAAGGUGUCTT; si-DAB2^#3^: sense: CCAGCAGUGAGAACUCAAATT; antisense: UUUGAGUUCUCACUGCUGGTT. Cells were transfected with si-RNA using Lipofectamine™ RNAiMAX (Invitrogen, 13778150, CA, USA) according to the manufacturer’s instructions. We choose si-DAB2^#1^ sequence and the virus packaging were performed by GenePharma (Suzhou, China). Cell transfection was performed following the handbook from the manufacturer. In brief, the cells were incubated in retroviral supernatant supplemented with 10 μg/mL polybrene (Solarbio, H8761). 24 h later, the medium was changed to a fresh complete medium. After 72 h infection, the cells were selected with 2.5 μg/mL puromycin (Solarbio, P8230) in the culture medium.

Conditioned media from HTR-8/SVneo (CM-HTR-8/SVneo^shNC^ and CM-HTR-8/SVneo^shDAB2^) were obtained following 48 h of culture. The collected media were kept in sterile tubes and centrifuged at 800 × g for 10 min, and the supernatants were stored at −80 °C and utilized within 1 month.

1. Immunohistochemistry and immunofluorescence

Tissues were fixed in 4% paraformaldehyde (Solarbio, P1110), dehydrated, embedded in paraffin, and sectioned. Sections were dewaxed, rehydrated, and treated with H_2_O_2_. After blocking with Bovine Serum Albumin (Thermo Fisher Scientific,37525), they were incubated with primary antibodies, followed by secondary antibodies. Staining was performed using diaminobenzidine and Hematoxylin (Beyotime, China). hVSMCs and HTR-8/SVneo cells were fixed and treated with TritonX100 (Beyotime, P0096). HTR-8/SVneo cells were incubated with DAB2 and Ki67 antibodies, followed by fluorescence-labeled secondary antibodies. hVSMCs were incubated with α-SMA antibodies and a fluorescence-labeled secondary antibody. DAPI (Sigma, 28718-90-3) was used for nuclear staining. Microscope images were captured using a Nikon microscope. Antibodies used are listed in Supplementary Table S3b.

1. RNA preparation and quantitative real-time PCR

Total RNA from the placenta tissues, cultured HTR-8/SVneo, and hVSMCs were extracted with TRIzol reagent (Invitrogen, 15596018) according to the manufacturer’s instruction. RNA purity and concentration were determined using a spectrometer (Thermo Fisher Scientific, Nanodrop 2000c). Reverse transcription was carried out by using the Oligo Primer (Shanghai Generay Biotech Co, Ltd.) and into complementary DNA (cDNA) using PrimeScriptTM RT Master Mix (TaKaRa, RR036A), and then cDNA was used as a template for quantitative real-time PCR with TB Green® Fast qPCR Mix (TaKaRa, RR430A) according to manufacturer’s instructions. The primer sequences are shown in Supplementary Table S3a. After the PCR reaction process, the relative expression of certain genes was normalized by GAPDH using the 2^−ΔΔCT^ method.

1. Protein preparation and Western blot analysis

Total protein from the placenta tissues, cultured hVSMCs and HTR-8/SVneo were extracted with RIPA lysis buffer (Thermo Fisher Scientific, #89901, USA), and 30-50 μg protein was subjected to 10% SDS-PAGE and subsequent electrotransfer to a 0.45μm pore size polyvinylidene fluoride (PVDF) membrane (Millipore, USA). ECL Western blotting substrate (Thermo Fisher Scientific, 32109) was applied for visualization. The relative density of the detected molecule was normalized by the value of GAPDH in the same blot. The intensity of protein bands was quantified using Image J (National Institutes of Health). All antibodies were listed in Supplementary Table S3b.

1. Wound healing assay

The medium was replaced with a serum-free medium containing 1 μg/ml mitomycin C (Sigma-Aldrich; Merck KGaA, St. Louis, USA) for 1 h when the cell culture was confluent. 10-μl pipette tips were used to establish the cell scratches. The cell surface was washed once with a serum-free medium to remove cell debris and observed under a microscope (IX53; Olympus Corporation, Japan). The different group cells were cultured for 24 h, 48 h, and 72 h, and images were obtained. Cell migration was determined following a comparison of the scratch width.

1. Transwell assay

The transwell chamber (Corning, USA) was placed in a 24-well plate. The cells (HTR-8/SVneo and hVSMCs) were washed with PBS and trypsinized. Subsequently, conditioned culture medium was added to create a single-cell suspension. Medium containing 2% FBS was added to the lower chamber. A total of 200 μl cell suspension (cell numbers were 2 x 104) was added to the upper chamber. The cells were incubated for 48 h and the transwell chambers were cleaned with PBS. The remained invasive cells were fixed with 4% polyformaldehyde for 20 min at room temperature. Crystalline purple (Amresco, Shanghai, China) solution (0.5%) was incubated for 10 min with the cells to stain them. The cells in the upper chamber were removed and invaded the lower microporous membrane were counted using an inverted microscope (IX53; Olympus Corporation, Japan). A total of 5 fields of each sample were counted, and the average value was obtained.

1. Flow cytometry

The apoptotic influence of different group CM of HTR-8/SVneo on hVSMCs still needs to tested. The cells were collected and incubated in a binding buffer, followed by staining with Annexin V-FITC and PI solution in an Annexin V-FITC Apoptosis Detection kit (Beyotime Biotech Inc, Shanghai, China). The apoptotic cells were measured by a flow cytometer (Life Technoligies, USA) and the apoptotic rate of cells apoptosis was presented as the percentage of cells with Annexin V-FITC staining positive and PI staining negative or positive.

1. The establishment of the human chorionic villous explants and placenta and decidua co-culture system

Villous explant cultures were established from first-trimester human placentae using the method of Caniggia et al [11]. Small fragments (15–20 mg wet weight) of placental villi from 6- 8 weeks gestation, were teased apart, and placed on the 24-well cultured dish precoatd with 100μl undiluted phenol Matrigel substrate (Corning, USA). Explant Media serum-free Dulbecco modified Eagle medium media (Sigma, USA) supplemented with 20% FBS (Corning, USA) and 100 lg/ml streptomycin (Sigma, St. Louis, MO), 100 U/ml penicillin.

The establishment of a placenta and decidua co-culture system was conducted as previously described [12]. Briefly, 200 μL Matrigel was added to the upper area of a 24-well co-culture chamber (pore size 8 μm, Corning, USA) and incubated at 37 °C for 1 h. Placenta and decidua samples were collected from 12 cases of human early pregnancy (6–8 weeks), the decidua tissue was cut into 1 × 1 cm2 patches and placed on solidified Matrigel. Then, the villous tissue tip part was cut into pieces and scattered on the decidua, and co-cultured with conditioned media at 37 °C for 6 days. The medium was changed every 2 days. After incubation, the decidua part was fixed with 4% paraformaldehyde, and immunofluorescence staining was performed.

1. Immunoprecipitation-Mass Spectrometry (IP-MS)

To investigate the protein interaction with DAB2, IP experiments were performed according to the manual of the Immunoprecipitation Kit with Protein A + G Magnetic Beads (Beyotime, P2179S). EVT Cell line HTR-8/SVneo was used to prepare the IP sample. After co-IP with DAB2-conjugated protein A + G magnetic beads, the beads-Ab-Ag complexes were resolved with SDS-PAGE. The discrete bands between DAB2 and IgG control were isolated, digested, purified, and subjected to liquid chromatograph (LC)-MS in Novegene Company (Guangzhou, China). Please refer to Supplementary Data 1 for detailed mass spectrometry results.

Reference

1. Roberts JM, Rich-Edwards JW, McElrath TF, Garmire L, Myatt L: Subtypes of Preeclampsia: Recognition and Determining Clinical Usefulness. Hypertension 2021, 77(5):1430-1441.

2. Brown MA, Magee LA, Kenny LC, Karumanchi SA, McCarthy FP, Saito S, Hall DR, Warren CE, Adoyi G, Ishaku S: Hypertensive Disorders of Pregnancy: ISSHP Classification, Diagnosis, and Management Recommendations for International Practice. Hypertension 2018, 72(1):24-43.

3. Pique-Regi R, Romero R, Tarca AL, Sendler ED, Xu Y, Garcia-Flores V, Leng Y, Luca F, Hassan SS, Gomez-Lopez N: Single cell transcriptional signatures of the human placenta in term and preterm parturition. Elife 2019, 8.

4. Chen J, Du L, Wang F, Shao X, Wang X, Yu W, Bi S, Chen D, Pan X, Zeng S et al: Cellular and molecular atlas of the placenta from a COVID-19 pregnant woman infected at midgestation highlights the defective impacts on foetal health. Cell Prolif 2022, 55(4):e13204.

5. Du L, Deng W, Zeng S, Xu P, Huang L, Liang Y, Wang Y, Xu H, Tang J, Bi S et al: Single-cell transcriptome analysis reveals defective decidua stromal niche attributes to recurrent spontaneous abortion. Cell Prolif 2021, 54(11):e13125.

6. Germain PL, Lun A, Garcia Meixide C, Macnair W, Robinson MD: Doublet identification in single-cell sequencing data using scDblFinder. F1000Res 2021, 10:979.

7. Vieira Braga FA, Kar G, Berg M, Carpaij OA, Polanski K, Simon LM, Brouwer S, Gomes T, Hesse L, Jiang J et al: A cellular census of human lungs identifies novel cell states in health and in asthma. Nat Med 2019, 25(7):1153-1163.

8. Xi NM, Li JJ: Benchmarking Computational Doublet-Detection Methods for Single-Cell RNA Sequencing Data. Cell Syst 2021, 12(2):176-194.e176.

9. Zhang Y, Liu W, Wu M, Li Q, Liu Y, Yang L, Chen Y, Zhong Y, Liu X, Zou L: PFKFB3 regulates lipopolysaccharide-induced excessive inflammation and cellular dysfunction in HTR-8/Svneo cells: Implications for the role of PFKFB3 in preeclampsia. Placenta 2021, 106:67-78.

10. Wu M, Liu W, Huang H, Chen Z, Chen Y, Zhong Y, Jin Z, Liu X, Zou L: PVT1/miR-145-5p/HK2 modulates vascular smooth muscle cells phenotype switch via glycolysis: The new perspective on the spiral artery remodeling. Placenta 2022, 130:25-33.

11. Caniggia I, Grisaru-Gravnosky S, Kuliszewsky M, Post M, Lye SJ: Inhibition of TGF-beta 3 restores the invasive capability of extravillous trophoblasts in preeclamptic pregnancies. J Clin Invest 1999, 103(12):1641-1650.

12. Wu D, Liu Y, Liu X, Liu W, Shi H, Zhang Y, Zou L, Zhao Y: Heme oxygenase-1 gene modified human placental mesenchymal stem cells promote placental angiogenesis and spiral artery remodeling by improving the balance of angiogenic factors in vitro. Placenta 2020, 99:70-77.

*Supplymentary table S1. Description of patient information of placental tissue origin for single-cell sequencing*

|  | Gestation-matched control 1^#^ | Gestation-matched control 2^#^ | Gestation-matched control 3^#^ |
| --- | --- | --- | --- |
| Age (years) | 30 | 30 | 33 |
| Gestation age(weeks) | 24w | 25w^+1^ | 32w^+4^ |
| Blood pressure (mm Hg) | 138/89 | 123/79 | 125/75 |
| Diagnosis | premature birth | premature birth | premature birth, cervical incompetence |
| Other Complications | NONE | NONE | NONE |

|  | Early-onset preeclampsia 1^#^ | Early-onset preeclampsia 2^#^ | Early-onset preeclampsia 3^#^ | Early-onset preeclampsia4^#^ |
| --- | --- | --- | --- | --- |
| Age (years) | 38 | 40 | 26 | 22 |
| Gestation age(weeks) | 28w^+5^ | 23w | 26w^+2^ | 27w^+5^ |
| Blood pressure (mm Hg) | 167/105 | 170/92 | 160/100 | 180/113 |
| Diagnosis | Sever preeclampsia, EOPE | Sever preeclampsia, EOPE | Sever preeclampsia, EOPE | Sever preeclampsia, EOPE |
| Gestational age at diagnosis of preeclampsia | 22 | 21 | 24 | 22 |
| Other Complications | NONE | NONE | NONE | NONE |

Supplymentary *table S2. Clinical characteristics of women included in the study*

1. Clinical characteristics of NC group (n=6)

|  | NC 1 | NC 2 | NC 3 | NC 4 | NC 5 | NC 6 |
| --- | --- | --- | --- | --- | --- | --- |
| Age (years) | 32 | 34 | 31 | 30 | 34 | 30 |
| Gestation age(weeks) | 25w+2 | 24w+3 | 32w+1 | 25w+3 | 29w+6 | 34w+1 |
| Blood pressure (mm Hg) | 107/69 | 110/70 | 108/80 | 110/72 | 104/69 | 110/80 |
| Diagnosis | premature birth, cervical incompetence | premature birth, cervical incompetence | premature birth, cervical incompetence | premature birth, cervical incompetence | premature birth, cervical incompetence | premature birth, cervical incompetence |
| Complications | NONE | NONE | NONE | NONE | NONE | NONE |

1. Clinical characteristics of EOPE(n=6)

|  | EOPE 1 | EOPE 2 | EOPE 3 | EOPE 4 | EOPE 5 | EOPE 6 |
| --- | --- | --- | --- | --- | --- | --- |
| Age (years) | 31 | 31 | 35 | 34 | 34 | 32 |
| Gestation age(weeks) | 28w+1 | 27w+3 | 27w+5 | 26w+6 | 25w+4 | 24w+3 |
| Blood pressure (mm Hg) | 167/107 | 163/109 | 187/130 | 163/110 | 162/101 | 161/101 |
| Diagnosis | EOPE | EOPE | EOPE | EOPE | EOPE | EOPE |
| Complications | NONE | NONE | NONE | NONE | NONE | NONE |

c. The differences of important indicators between the two groups were statistically analyzed

|  | normal (n=6) | EOPE(n=6) | *p* |
| --- | --- | --- | --- |
| Age (years) | 32±0.098 | 33±1.673 | 0.3828 |
| Gestation age (weeks) | 28.17±4.167 | 29±1.789 | 0.6622 |
| Systolic pressure (mm Hg) | 108.2±2.401 | 167.2±9.928 | ＜0.0001 |
| Diastolic pressure (mm Hg) | 73.33±5.279 | 109.7±4.364 | ＜0.0001 |

d. Information on clinical samples used to examine DAB2 expression in decidua basalis tissues throughout pregnancy

| Gestation | 6-7 (n=6) | 8-9 (n=6) | 28-32 (n=6) | 37-39 (n=6) |
| --- | --- | --- | --- | --- |
| Ages(y) | 25~30 | 21~30 | 27~32 | 23~30 |
| Blood pressure (mm Hg) | 118.4±3.1 | 105.2±2.78 | 115.2±4.2 | 112±3.6 |
| Complications | NONE | NONE | NONE | NONE |

The student’s two-tailed t-test was used to statistically compare groups for each variable, unless otherwise denoted.

*Data are given as mean ± standard deviation.

*Supplementary table S3. a. qRT-PCR primer sequences*

| Genes |  | Primer Sequence (5’-3’) |
| --- | --- | --- |
| *DAB2* | F | ATCCTGATCCTTTCCGTGAC |
|  | R | TCAGCGGAGTAGACGAGCTA |
| *MMP2* | F | TGATCTTGACCAGAATACCATCGA |
|  | R | GGCTTGCGAGGGAAGAAGTT |
| *MMP9* | F | TCTGCCTGCACCACCGACG |
|  | R | CTGGGTGTAGAGTCTCTCG |
| *α-SMA* | F | AAAAGACAGCTACGTGGGTGA |
|  | R | GCCATGTTCTATCGGGTACTTC |
| *SM22α* | F | ATCCCAACTGGTTCCCTAAGAA |
|  | R | CCCATCTGTAACCCGATCACG |
| *Calponin* | F | CTGTCAGCCGAGGTTAAGAAC |
|  | R | GAGGCCGTCCATGAAGTTGTT |
| *MYH11* | F | CGCCAAGAGACTCGTCTGG |
|  | R | TCTTTCCCAACCGTGACCTTC |
| *GAPDH* | F | GAAGGTGAAGGTCGGAGTC |
|  | R | GAAGATGGTGATGGGATTTCC |
| *IL20R* | F | AATAACCAGATGCAGCCCATGT |
|  | R | CCGAAATGCAACTGTCCTCAC |
| *CCL20* | F | CTGCTTTGATGTCAGTGCTGC |
|  | R | TGCTGACCTAGGCTTGATGA |
| *IL1A* | F | GGTTGAGTTTAAGCCAATCCA |
|  | R | TCACCCAAGTCTGTTTTGG |
| *CXCL2* | F | GCTTGTCTCAACCCCGCATC |
|  | R | TGGATTTGCCATTTTTCAGCATCTT |
| *CXCL1* | F | CTCGAGGCCCCTGGGGCAGAAGCCTC |
|  | R | GATATCGGGGCTCAGCAGGCGGGTCT |
| *CXCL6* | F | TGCGTTGCACTTGTTTACGC |
|  | R | CGTTCTTCAGGGAGGCTACCA |
| *CXCL3* | F | CGCCCAAACCGAAGTCATAG |
|  | R | GCTCCCCTTGTTCAGTATCTTTT |
| *TGFA* | F | AGCTGCTAGCGCCTAGCGAT |
|  | R | CCCGTCTGATAGCGCATTCGTGT |
| *IL21R* | F | CGTGGGAGTCAGCATGCC |
|  | R | TGTCGTCGGCCATGAAGTG |

b. The information of Antibodies

| Antibodies | Source | Identifier | Application |
| --- | --- | --- | --- |
| primary antibodies |  |  |  |
| DAB2 | Abcam | Cat# ab76253 | IHC (1:500);  IF (1:500);  WB (1:1000) |
| α-SMA | Proteintech | Cat No.23660-1-AP | IF (1:500)  WB (1:1000) |
| CD31 | CST | 3528S | IF (1:500) |
| Ki67 | CST | 9129S | IF (1:500) |
| MYH-11 | Abcam | Cat# ab53219 | WB (1:1000) |
| CXCL8 | Abcam | Cat# ab289967 | IF (1:500) |
| MMP2 | CST | #4022S | WB (1:1000) |
| MMP9 | CST | #3852S | WB (1:1000) |
| AKT | CST | #4691S | WB (1:1000) |
| p-AKT | CST | #4060S | WB (1:1000) |
| p38 | CST | #9212S | WB (1:1000) |
| p-p38 | CST | #4511S | WB (1:1000) |
| Erk1/2 | CST | #9102S | WB (1:1000) |
| p-Erk1/2 | CST | #4370S | WB (1:1000) |
| JNK | CST | #9252S | WB (1:1000) |
| p-JNK | CST | #4668S | WB (1:1000) |
| GEF-H1 | Abcam | Cat# ab155785 | WB (1:1000) |
| GAPDH | Proteintech | Cat No. 60004-1-Ig | WB (1:5000) |
| Second antibody |  |  |  |
| Anti-rabbit IgG, HRP-linked | CST | #7074S | WB (1:10000) |
| Anti-mouse lgG, HRP-linked | CST | #7076S | WB (1:10000) |
| Anti-rabbit IgG, (Alexa Fluor® 488 Conjugate) | CST | #4412S | IF (1:500) |
| Anti-mouse IgG (Alexa Fluor® 594 Conjugate) | CST | #8890S | IF (1:500) |
