# Supplementary material for: Identification of the role of DAB2 and CXCL8 in uterine spiral artery remodeling in early-onset preeclampsia
Source: Cell Mol Life Sci. 2024 Apr 13;81(1):180. doi: 10.1007/s00018-024-05212-4 (PMC11016014; doi:10.1007/s00018-024-05212-4)
Supplement: Supplementary file 3 — Supplementary file3 (PDF 396462 KB) [file 18_2024_5212_MOESM3_ESM.pdf]

Fig.S1

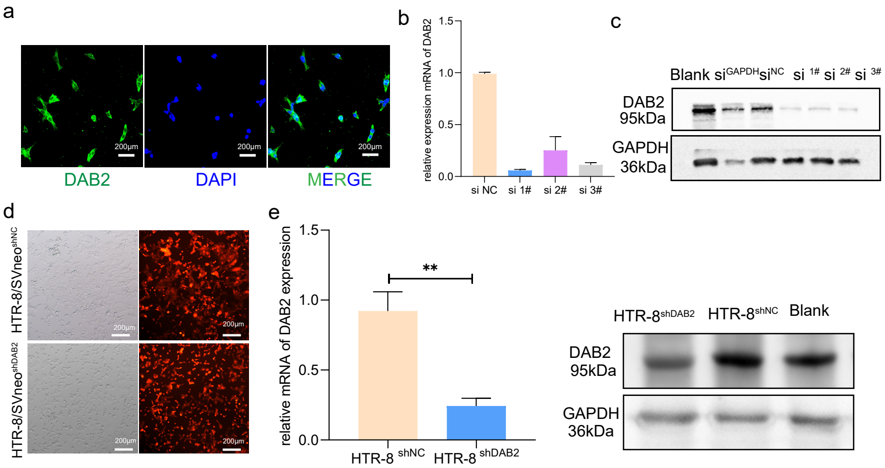
a
200μm
DAB2
DAPI
MERGE
b
relative expression mRNA of DAB2
1.5
1.0
0.5
0.0
si NC
si 1#
si 2#
si 3#
c
Blank si GAPDH si NC si 1# si 2# si 3#
DAB2
95kDa
GAPDH
36kDa
d
HTR-8/SVneo shNC
HTR-8/SVneo shDAB2
e
relative mRNA of DAB2 expression
**
HTR-8 shNC
HTR-8 shDAB2
HTR-8 shDAB2 HTR-8 shNC Blank
DAB2
95kDa
GAPDH
36kDa

Fig. S2

a

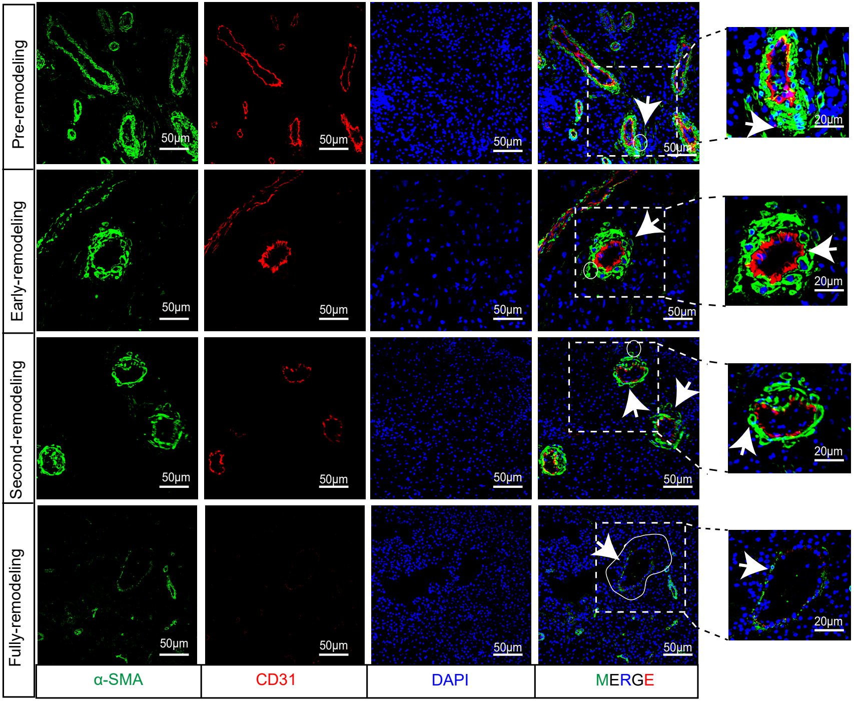

Pre-remodeling
Early-remodeling
Second-remodeling
Fully-remodeling
50μm
20μm
α-SMA
CD31
DAPI
MERGE

Fig. S3

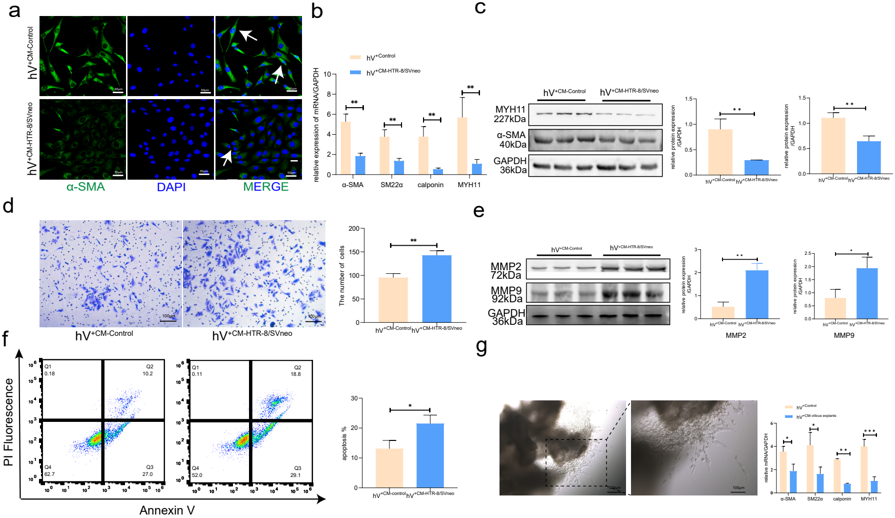

a
hV+CM-Control
hV+CM-HTR-8/SVneo
α-SMA
DAPI
MERGE
b
hV+Control
hV+CM-HTR-8/SVneo
relative expression of mRNA/GAPDH
α-SMA
SM22α
calponin
MYH11
c
hV+CM-Control
hV+CM-HTR-8/SVneo
MYH11 227kDa
α-SMA 40kDa
GAPDH 36kDa
relative protein expression /GAPDH
d
The number of cells
hV+CM-Control
hV+CM-HTR-8/SVneo
e
MMP2 72kDa
MMP9 92kDa
GAPDH 36kDa
MMP2
MMP9
f
PI Fluorescence
Annexin V
Q1 0.18
Q2 10.2
Q4 62.7
Q3 27.0
Q1 0.11
Q2 18.8
Q4 52.0
Q3 29.1
apoptosis %
hV+CM-control
hV+CM-HTR-8/SVneo
g
hV+Control
hV+CM-villous explants
relative mRNA/GAPDH
α-SMA
SM22α
calponin
MYH11

Fig. S4

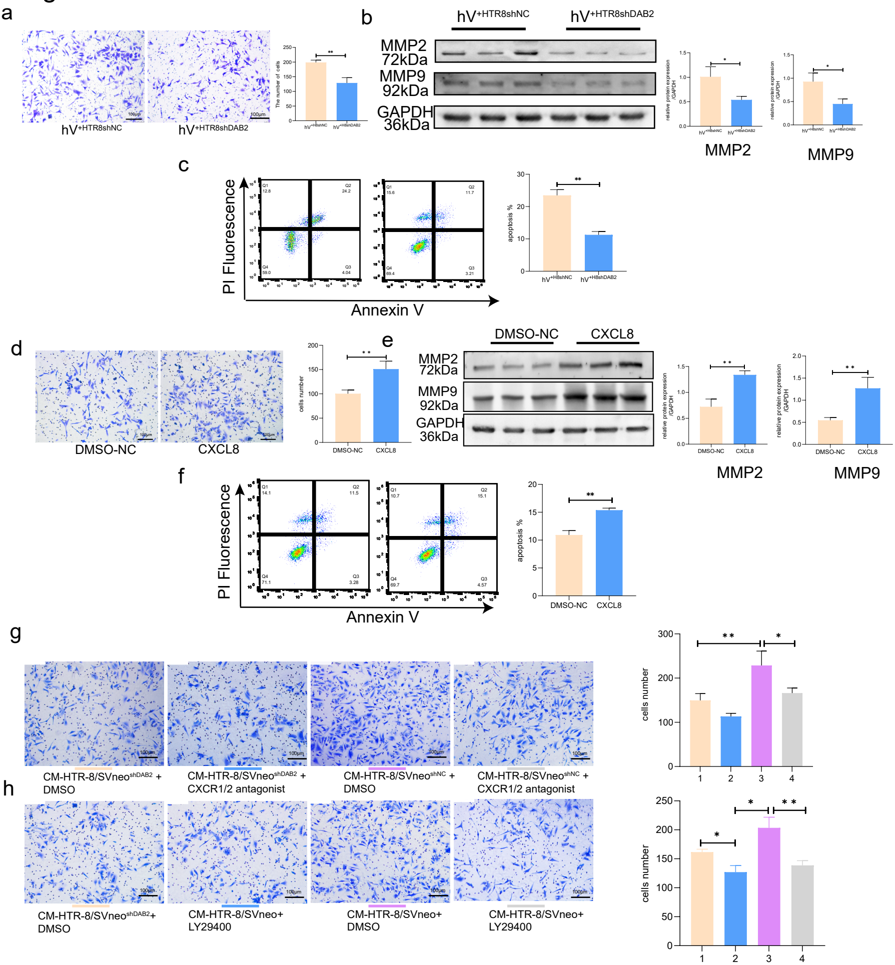

a
hV+HTR8shNC
hV+HTR8shDAB2
b
MMP2 72kDa
MMP9 92kDa
GAPDH 36kDa
MMP2
MMP9
c
PI Fluorescence
Annexin V
apoptosis %
d
DMSO-NC
CXCL8
cells number
e
f
g
h
CM-HTR-8/SVneoshDAB2 + DMSO
CM-HTR-8/SVneoshDAB2 + CXCR1/2 antagonist
CM-HTR-8/SVneoshNC + DMSO
CM-HTR-8/SVneoshNC + CXCR1/2 antagonist
CM-HTR-8/SVneoshDAB2+ DMSO
CM-HTR-8/SVneo+ LY29400
CM-HTR-8/SVneo+ DMSO
CM-HTR-8/SVneo+ LY29400

Fig.S5

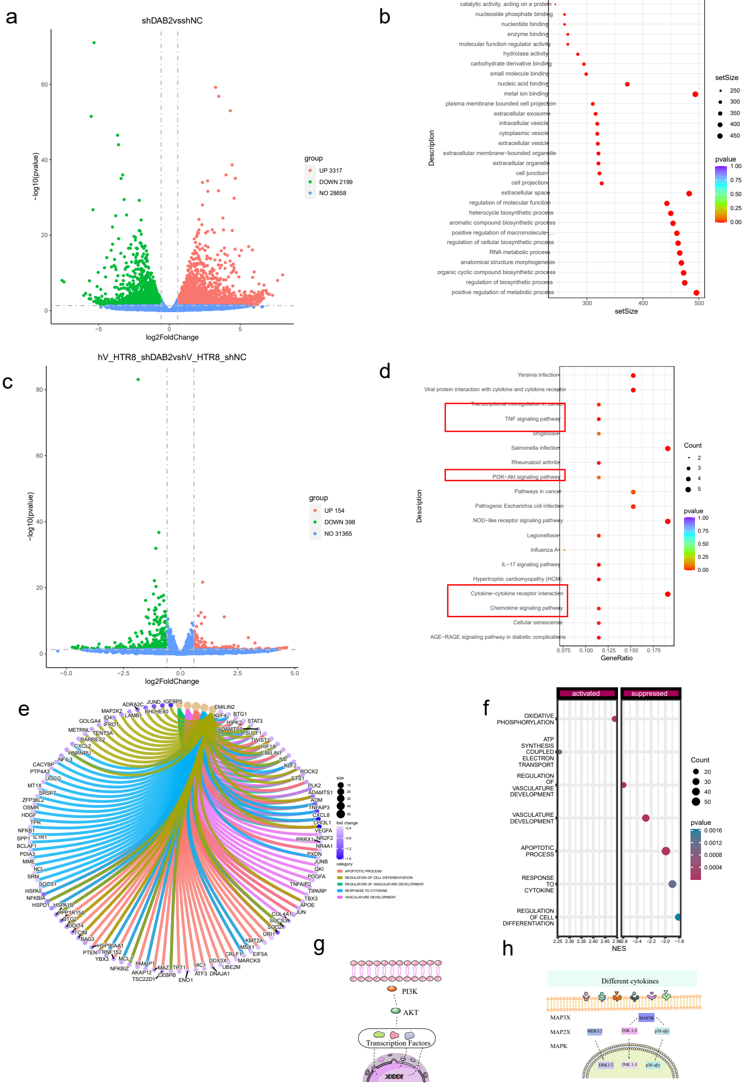
a
shDAB2vsshNC
-log10(pvalue)
log2FoldChange
group
UP 3317
DOWN 2199
NO 28658
b
catalytic activity, acting on a protein
nucleoside phosphate binding
nucleotide binding
enzyme binding
molecular function regulator activity
hydrolase activity
carbohydrate derivative binding
small molecule binding
nucleic acid binding
metal ion binding
plasma membrane bounded cell projection
extracellular exosome
intracellular vesicle
cytoplasmic vesicle
extracellular vesicle
extracellular membrane-bounded organelle
extracellular organelle
cell junction
cell projection
extracellular space
regulation of molecular function
heterocycle biosynthetic process
aromatic compound biosynthetic process
positive regulation of macromolecule-...
regulation of cellular biosynthetic process
RNA metabolic process
anatomical structure morphogenesis
organic cyclic compound biosynthetic process
regulation of biosynthetic process
positive regulation of metabolic process
Description
setSize
pvalue
c
hV_HTR8_shDAB2vshV_HTR8_shNC
-log10(pvalue)
log2FoldChange
group
UP 154
DOWN 398
NO 31365
d
Yersinia infection
Viral protein interaction with cytokine and cytokine receptor
Transcriptional misregulation in cancer
TNF signaling pathway
Shigellosis
Salmonella infection
Rheumatoid arthritis
PI3K-Akt signaling pathway
Pathways in cancer
Pathogenic Escherichia coli infection
NOD-like receptor signaling pathway
Legionellosis
Influenza A
IL-17 signaling pathway
Hypertrophic cardiomyopathy (HCM)
Cytokine-cytokine receptor interaction
Chemokine signaling pathway
Cellular senescence
AGE-RAGE signaling pathway in diabetic complications
Description
GeneRatio
Count
pvalue
e
size
fold change
category
APOPTOTIC PROCESS
REGULATION OF CELL DIFFERENTIATION
REGULATION OF VASCULATURE DEVELOPMENT
RESPONSE TO CYTOKINE
VASCULATURE DEVELOPMENT
f
activated
suppressed
OXIDATIVE PHOSPHORYLATION
ATP SYNTHESIS COUPLED ELECTRON TRANSPORT
REGULATION OF VASCULATURE DEVELOPMENT
VASCULATURE DEVELOPMENT
APOPTOTIC PROCESS
RESPONSE TO CYTOKINE
REGULATION OF CELL DIFFERENTIATION
NES
Count
pvalue
g
PI3K
AKT
Transcription Factors
h
Different cytokines
MAP3X
MAP2X
MAPK
MAP3K
MEK1/2
JNK 1-3
ERK1/2

Fig. S6

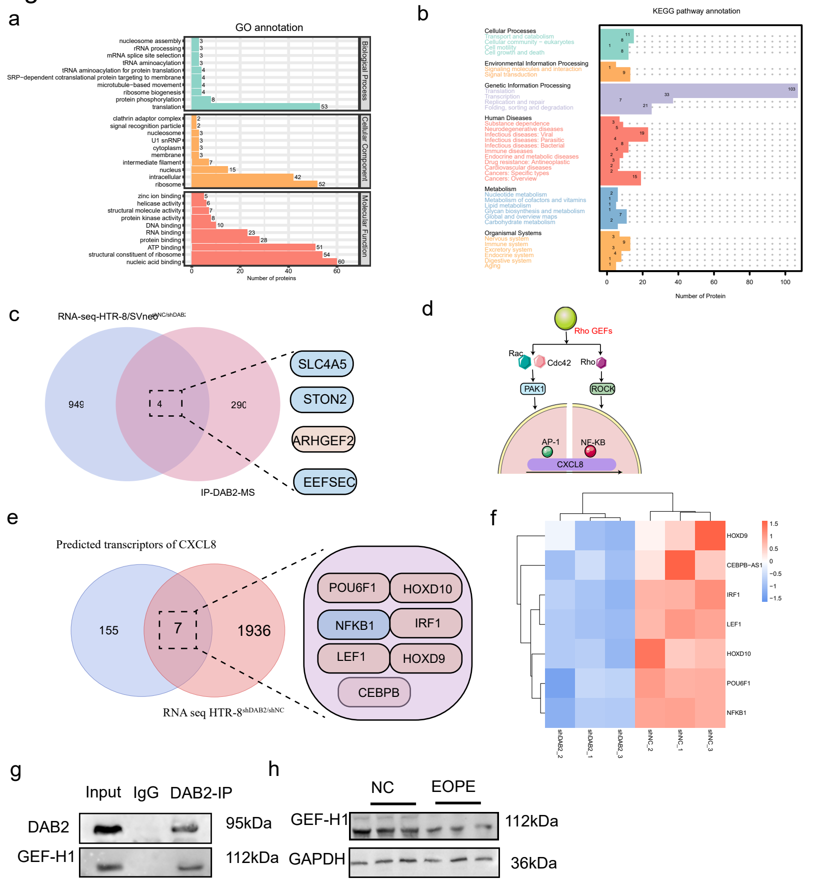

a
GO annotation
nucleosome assembly
rRNA processing
mRNA splice site selection
tRNA aminoacylation
tRNA aminoacylation for protein translation
SRP−dependent cotranslational protein targeting to membrane
microtubule−based movement
ribosome biogenesis
protein phosphorylation
translation
Biological Process
clathrin adaptor complex
signal recognition particle
nucleosome
U1 snRNP
cytoplasm
membrane
intermediate filament
nucleus
intracellular
ribosome
Cellular Component
zinc ion binding
helicase activity
structural molecule activity
protein kinase activity
DNA binding
RNA binding
protein binding
ATP binding
structural constituent of ribosome
nucleic acid binding
Molecular Function
Number of proteins
b
KEGG pathway annotation
Cellular Processes
Transport and catabolism
Cellular community − eukaryotes
Cell motility
Cell growth and death
Environmental Information Processing
Signaling molecules and interaction
Signal transduction
Genetic Information Processing
Translation
Transcription
Replication and repair
Folding, sorting and degradation
Human Diseases
Substance dependence
Neurodegenerative diseases
Infectious diseases: Viral
Infectious diseases: Parasitic
Infectious diseases: Bacterial
Immune diseases
Endocrine and metabolic diseases
Drug resistance: Antineoplastic
Cardiovascular diseases
Cancers: Specific types
Cancers: Overview
Metabolism
Nucleotide metabolism
Metabolism of cofactors and vitamins
Lipid metabolism
Glycan biosynthesis and metabolism
Global and overview maps
Carbohydrate metabolism
Organismal Systems
Nervous system
Immune system
Excretory system
Endocrine system
Digestive system
Aging
Number of Protein
c
RNA-seq-HTR-8/SVneo shNC/shDAB2
949
4
290
IP-DAB2-MS
SLC4A5
STON2
ARHGEF2
EEFSEC
d
Rho GEFs
Rac
Cdc42
Rho
PAK1
ROCK
AP-1
NF-KB
CXCL8
e
Predicted transcriptors of CXCL8
155
7
1936
RNA seq HTR-8 shDAB2/shNC
POU6F1
HOXD10
NFKB1
IRF1
LEF1
HOXD9
CEBPB
f
HOXD9
CEBPB−AS1
IRF1
LEF1
HOXD10
POU6F1
NFKB1
shDAB2_2
shDAB2_1
shDAB2_3
shNC_2
shNC_1
shNC_3
g
Input
IgG
DAB2-IP
DAB2
GEF-H1
95kDa
112kDa
h
NC
EOPE
GEF-H1
GAPDH
112kDa
36kDa
